# Rapid northern hemisphere ice sheet melting during the penultimate deglaciation

**DOI:** 10.1038/s41467-022-31619-3

**Published:** 2022-07-02

**Authors:** Heather M. Stoll, Isabel Cacho, Edward Gasson, Jakub Sliwinski, Oliver Kost, Ana Moreno, Miguel Iglesias, Judit Torner, Carlos Perez-Mejias, Negar Haghipour, Hai Cheng, R. Lawrence Edwards

**Affiliations:** 1 Department of Earth Sciences, ETH Zürich, Zurich, Switzerland; 2 Grup de Recerca Consolidat en Geociències Marines, Department de Dinàmica de la Terra i de l’Oceà, Universitat de Barcelona, Barcelona, Spain; 3 School of Geographical Sciences, University of Bristol, Bristol, UK; 4 Earth and Environmental Sciences, University of Exeter, Exeter, UK; 5 Currently at School of Earth and Environmental Sciences, University of St. Andrews, St. Andrews, UK; 6 Department of Geoenvironmental Processes and Global Change, Pyrenean Institute of Ecology-CSIC, Zaragoza, Spain; 7 Department of Geology, University of Oviedo, Oviedo, Spain; 8 Institute of Global Environmental Change, Xi’an Jiaotong University, Xi’an, China; 9 Laboratory for Ion Beam Physics, Department of Physics, ETH Zurich, Switzerland; 10 Department of Earth and Environmental Sciences, University of Minnesota, Minneapolis, USA; 11 School of Geography, Nanjing Normal University, Nanjing, 210023 China

**Keywords:** Palaeoclimate, Palaeoceanography

## Abstract

The rate and consequences of future high latitude ice sheet retreat remain a major concern given ongoing anthropogenic warming. Here, new precisely dated stalagmite data from NW Iberia provide the first direct, high-resolution records of periods of rapid melting of Northern Hemisphere ice sheets during the penultimate deglaciation. These records reveal the penultimate deglaciation initiated with rapid century-scale meltwater pulses which subsequently trigger abrupt coolings of air temperature in NW Iberia consistent with freshwater-induced AMOC slowdowns. The first of these AMOC slowdowns, 600-year duration, was shorter than Heinrich 1 of the last deglaciation. Although similar insolation forcing initiated the last two deglaciations, the more rapid and sustained rate of freshening in the eastern North Atlantic penultimate deglaciation likely reflects a larger volume of ice stored in the marine-based Eurasian Ice sheet during the penultimate glacial in contrast to the land-based ice sheet on North America as during the last glacial.

## Abstract (web summary)

Stalagmites from NW Iberia record the rapid demise of large ice sheets during the penultimate deglaciation, and reveal decadal-scale feedbacks between warming and ice melting.

## Introduction

During glacial terminations, retreating ice sheets release large meltwater fluxes into the ocean. This leads to rising sea level and can initiate strong climate feedbacks when sufficiently large meltwater fluxes reach regions of deepwater formation in the North Atlantic. These ocean-atmosphere interactions, together with associated ocean CO_2_ release, operate as strong and rapid amplifiers of the original orbital induced insolation change. The rapid retreat of ice sheets which yields high meltwater fluxes, can be caused by marine ice sheet instability, ice sheet saddle collapse and marine ice cliff failure^1,2^. While the timing and associated deglacial feedbacks have been extensively studied for the last deglaciation (Termination I, TI)^3^, knowledge of the insolation thresholds, rate of ice sheet melting, and feedback sequence of previous terminations is more limited due to the lack of direct absolute chronology for ice retreat and deglacial warming. Some studies suggest that the sequence of millennial feedbacks may be different in previous terminations^4,5^. The penultimate deglaciation, Termination II (TII), is of particular interest because orbital boundary conditions were different, and the termination was followed by an interglacial with a + 1.2 to 5.3 m sea level highstand - suggesting both Greenland and Antarctic ice sheets retreated further than during TI^6^, despite similar highs in atmospheric CO_2_^7^. To fully understand the last interglacial highstand, clear knowledge of the ice sheets melting time and retreat rates during TII is needed^8,9^.

**Fig. 1:**
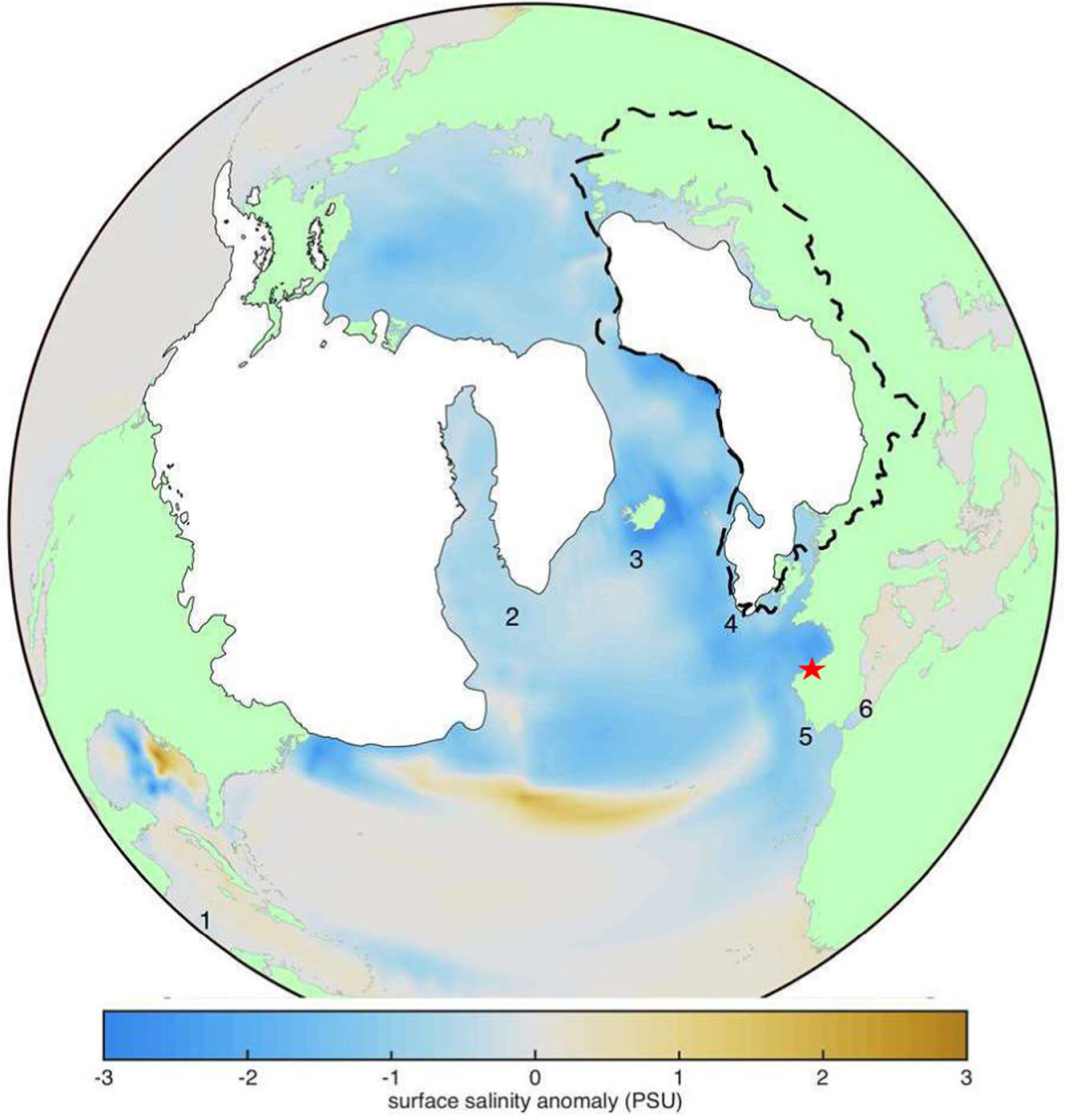
Northern hemisphere ice sheets and site locations. The distribution of North American Ice Sheet (NAIS) and Eurasian Ice Sheet (EIS) during the LGM (white coverage, after^108^) and the maximum extent of EIS glacial deposits prior to the last glacial cycle (black dashed lines, after^46^). Color scale over the ocean depicts the distribution of marine salinity anomalies at 16 ka resulting from a 3000 ky simulation with meltwater derived 45% from the EIS and 55% from the NAIS delivered to appropriate coastal outlets and leading to a 20% AMOC slowdown^10^. NW Iberian cave site (red star), and locations discussed: 1 Western Caribbean Site (ODP 999); 2 Eirik drift (MD03-2664); 3 North Atlantic Sites (ODP 983 and 984); 4 Irish Margin site (MD01-2461); 5 W Iberian Margin MD99-2334K; MD01-2343; MD01-2444; MD95-2042, MD95-2040; SHAK06-5K); 6 S Iberian Margin (ODP 976 and 977; MD95-2043).

Water stored in high latitude ice sheets has a δ^18^O lower than the mean ocean, meaning the deglacial melting of the Northern Hemisphere (NH) ice sheets lowers the δ^18^O of the surface ocean (δ^18^O_sw_). For regions proximal to release of glacial meltwater, such as the North Atlantic, the rate of δ^18^O_sw_ depletion and freshening can exceed the global average^10,11^, which has been useful in diagnosing meltwater routes during TI^12–14^. We propose that the evolution of the North Atlantic δ^18^O_sw_ can be recorded in coastal European speleothems through the transfer of the δ^18^O_sw_ signal of the ocean moisture source to the δ^18^O of rainfall, and thereby the δ^18^O of dripwater^15^ from which the speleothem is formed. We report on the North Iberian Speleothem Archive (NISA) from caves within 10 km of the Atlantic coast (Fig. 1) at coastal elevation (<70 m above sea level), directly adjacent to the main moisture source region in the eastern North Atlantic. We employ speleothems spanning the last 25 ky to evaluate the δ^18^O and δ^13^C proxy relationships against independent records of regional temperature and δ^18^O_sw_. The analysis of speleothems covering TI and TII from the same caves and same proxy indicators provides, by the first time, the opportunity to unambiguously compare the timing and rate of the last two deglaciations on an absolute ^230^Th chronology, with rates further refined by annual layer counting in TII. We show that the NISA record provides a unique opportunity to directly link the impact of rapid freshening of the North Atlantic on regional atmospheric temperatures, using the same archive, in order to test mechanisms for deglacial feedbacks.

## Results and discussion

### Controls on the δ^18^O and δ^13^C of NISA speleothems

Using six stalagmites spanning the last 25 ky to generate a composite splice speleothem δ^18^O record (Methods; Supplementary Figs. 1 and 2), we test the relationship between the speleothem δ^18^O and independent δ^18^O_sw_ estimates from foraminifera in North Atlantic marine sediment cores (Supplementary Fig. 3).

**Fig. 2:**
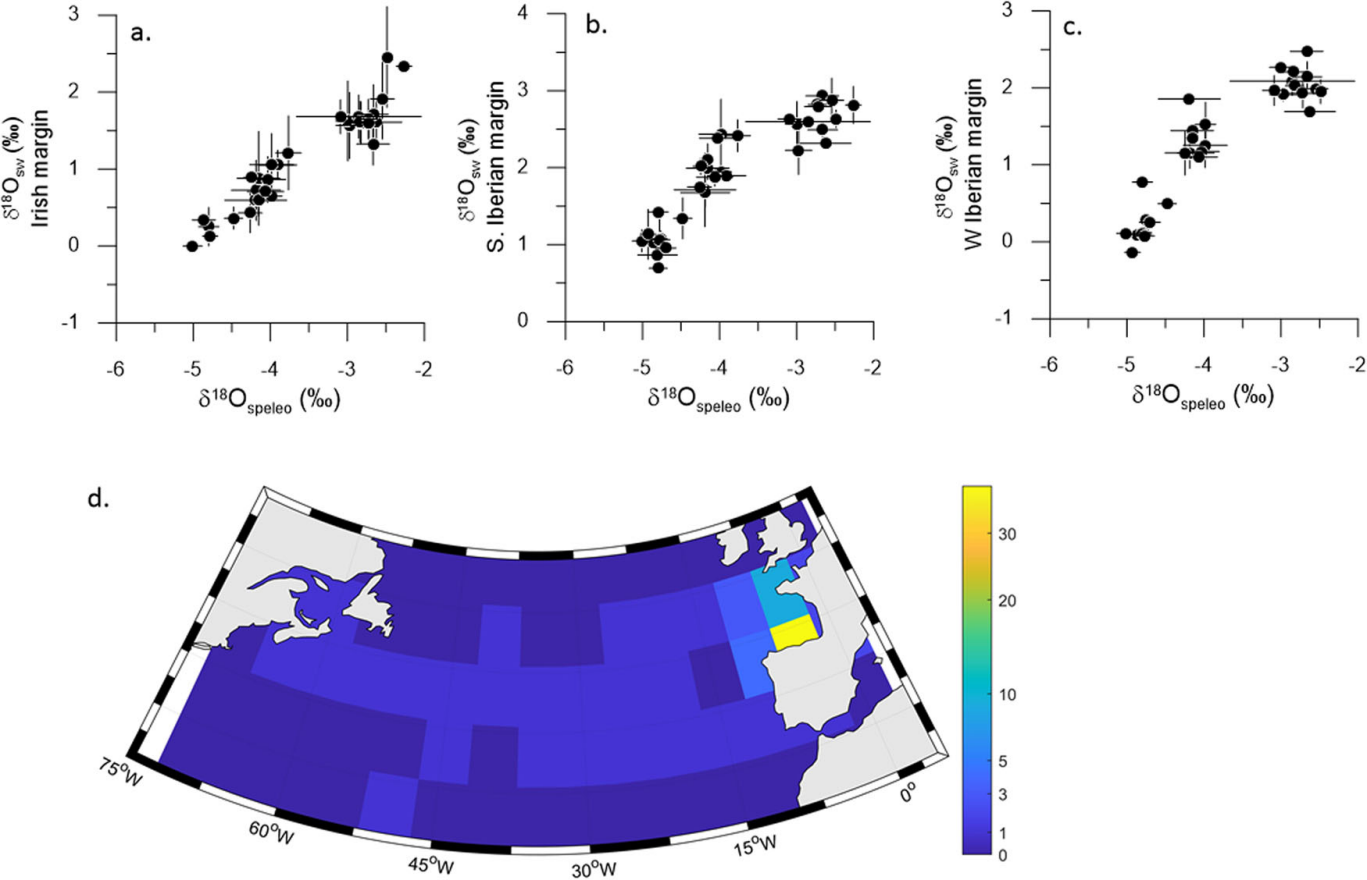
Prediction of δ^18^O_sw_ from δ^18^O_NISA_. **a**–**c** δ^18^O_sw_ vs δ^18^O_NISA_ in fixed 500 ky bins over the time interval from 25 to 5 ka BP, on original marine chronologies. Horizontal and vertical lines illustrate plus and minus one standard deviation of the δ^18^O data to illustrate the range of variation within each age bin. Results show δ^18^O_sw_ from the a) Irish margin^16,32^, r^2^ = 0.91. **b** W. Iberian margin^18^, r^2^ = 0.85. **c** S. Iberian margin^17^ r^2^ = 0.85. **d** Proportion (as %) of oceanic moisture recharge events originating in each 5° × 5°grid over the ocean, for precipitation events 2015–2016 near the cave location, as described in Methods. For regressions, all *p*-values are < 0.001; full data in Supplementary Table 1.

We find that throughout the last 25 ka, where both marine and speleothem records rely on independent absolute chronologies, δ^18^O_NISA_ exhibits an unusually close correlation with the δ^18^O_sw_ in the eastern North Atlantic Ocean. The δ^18^O_NISA_ is most strongly correlated (r^2^ of 0.91) with the δ^18^O_sw_ on the Irish Margin^16^ and a low standard error indicates a consistent relationship throughout the deglaciation despite the potential for variation in atmospheric and surface ocean boundary conditions (Fig. 2, Supplementary Table 1). Strong correlation is also attained for the δ^18^O_sw_ of the S. Iberian Margin^17^ and W. Iberian Margin^18^ (Fig. 2, Supplementary Table 1), as expected given the southward flowing eastern boundary current from the Irish margin along the Iberian margin and via the Atlantic Jet surface water flow through the Straits of Gibraltar^19^. The latter regions δ^18^O_sw_ are also closely correlated with the Irish margin δ^18^O_sw_ (Supplementary Table 2) and show no systematic changes in the δ^18^O_sw_ gradients (Supplementary Fig. 3). A HadCM3 model of the early phases of the TI deglaciation, simulating meltwater based on ICE6G ice sheet evolution and drainage routing (meltwater ~45% from EIS and 55% from the NAIS), yields similar spatial pattern of meltwater- induced salinity anomaly. Low salinity is concentrated in the North Atlantic north of 40°N, including the Irish Margin^10^ and extending further southward due to eastern boundary current^20^ along the Iberian Peninsula, NW coast of Africa, and into the Alboran Sea (Fig. 1). The strong relationship between δ^18^O_NISA_ and the proximal surface ocean δ^18^O_sw_ is not observed in Mediterranean region speleothems over TI (Supplementary Table 3), likely due to additional hydrological effects^21–23^.

Several factors may contribute to the coherence between δ^18^O_NISA_ and the δ^18^O_sw_ of the proximal ocean. Our analysis of all precipitation events in Northern Spain at a station <100 km west of the cave site in 2015–2016, shows that the majority of rainfall events in this region derive from oceanic moisture uptake in the easternmost North Atlantic ocean proximal to the Iberian Peninsula, especially north of Iberia (Fig. 2d). The high east-west topographic barrier of the Pyrenees and Cantabrian mountains of Northern Spain likely contributes both to the modern dominance of this northern proximal ocean moisture source and the stability of this moisture source over time. The location of caves at coastal elevation, directly adjacent to the main Atlantic moisture source region, minimizes the isotopic distillation between the moisture source area and the cave site. The imprint of such distillation on speleothem δ^18^Ofrom European caves increases with their increasing inland distance and elevation^24^; and temperature modulates this distillation so that temperature rather than the δ^18^O_sw_ of moisture source, becomes the dominant influence on δ^18^O_NISA_ in central Europe^25,26^. In our NW Iberian cave locations, rainfall monitoring shows that the slight decrease in rainfall δ^18^O with decreasing temperature^27^ appears of similar magnitude but opposite sign as the temperature-dependent fractionation between dripwater and calcite in the cave, leaving the δ^18^O_sw_ of the Atlantic moisture source as the principal variable expressed in the stalagmites. We propose that the δ^18^O_sw_ -δ^18^O_NISA_ relationship identified in TI remained stable over TII.

**Fig. 3:**
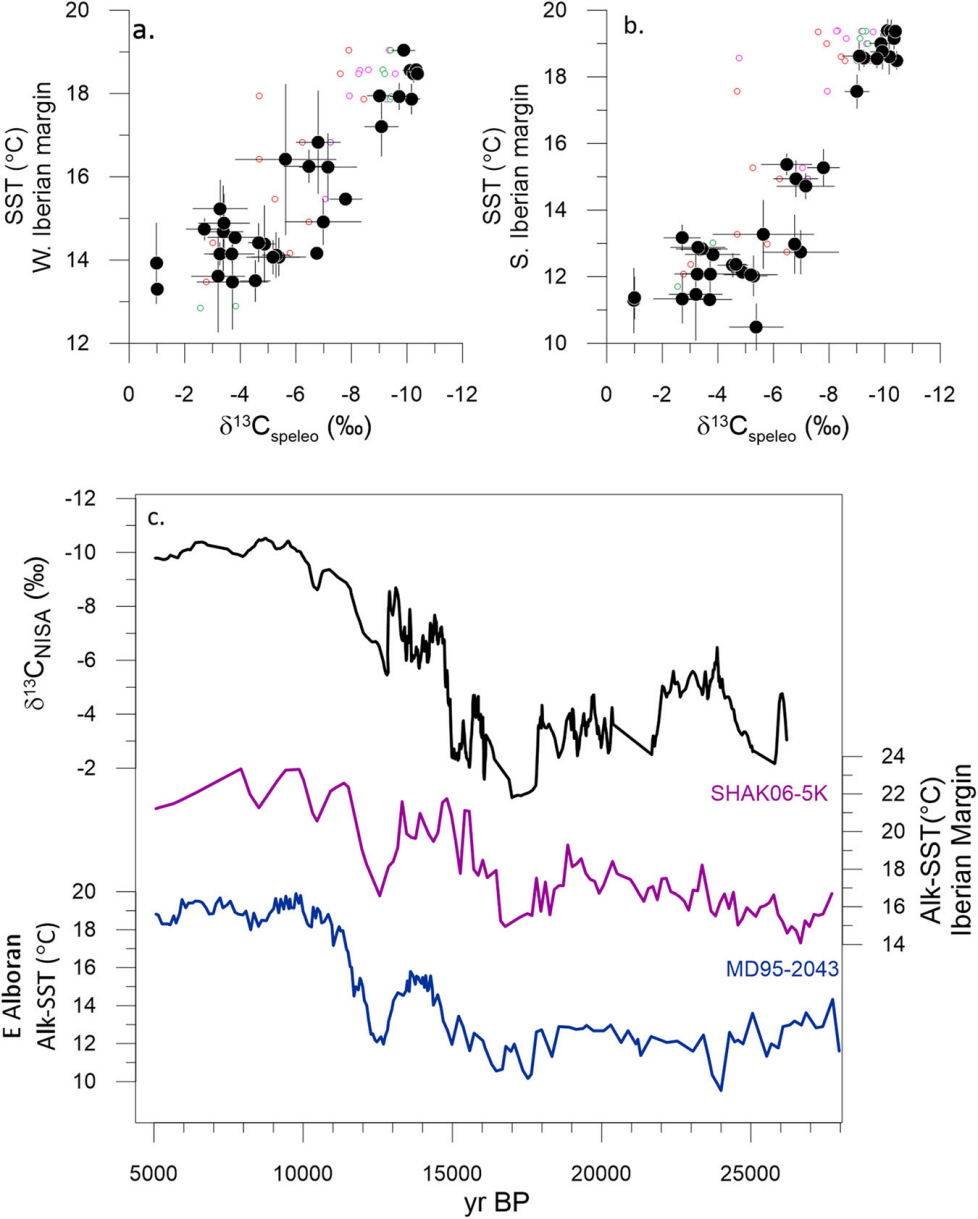
Relationship between regional temperature and NW Iberian speleothem δ^13^C_NISA_ over TI. **a**, **b** The scatterplots show δ^13^C_NISA_ for fixed time bins (500 years) from stalagmites Candela, Galia, Laura, and Alicia, vs alkenone SST from the S Iberian margin^95^ and W Iberian Margin^29^ respectively. Black symbols denote the average for the splice, and small colored symbols denote stalagmite increments where they do not comprise the splice, from Candela (red), Laura (brown, Galia (green) and Alicia (fuchsia). For the S. Iberian margin, *r*^2^ = 0.87 and *p* « 0.01. For the W. Iberian margin, *r*^2^ = 0.82 and *p* << 0.01. **c** Time series of the NISA δ^13^C_NISA_ splice, and the alkenone SST from the MD95-2043 on S Iberian margin^95^ and SHAK06-5K on W Iberian Margin^29^.

Additionally, we confirm that over TI, the δ^13^C_NISA_ in stalagmites from our caves varies inversely with regional temperature records from marine archives (Methods, Fig. 3; Supplementary Fig. 4), matching millennial variations in SST despite potential deviations in marine ^14^C chronology due to variation in surface ocean reservoir ages^28^ and the potential for alkenones of differing production ages to be deposited together^29^. Similar temporal correlation between temperature and δ^13^C during marine isotope stages 3 and 4 has been observed in speleothems from the Atlantic coastal region of France^30^. We conclude that the main trends are attributable to the carbon isotope signature acquired through rain equilibration with soil gas and bedrock dissolution, rather than in-cave processes such as prior calcite precipitation (Methods). Multiproxy process modeling suggests that this correlation arises because higher temperature strongly increases vegetation productivity in this biogeographic regime, enhancing soil CO_2_ production and oversupply of CO_2_ to karst waters, both factors which produce more negative δ^13^C in speleothems (δ^13^C_speleo_)^31^. We infer that the specific slope of the temperature vs δ^13^C_NISA_ relationships may be specific to a given cave systems and host lithology, and may be sensitive to the degree of smoothing of millennial-scale changes which is affected by the speleothem growth rate and the years aggregated in each drilling increment.

### NISA speleothem records spanning TII

**Fig. 4:**
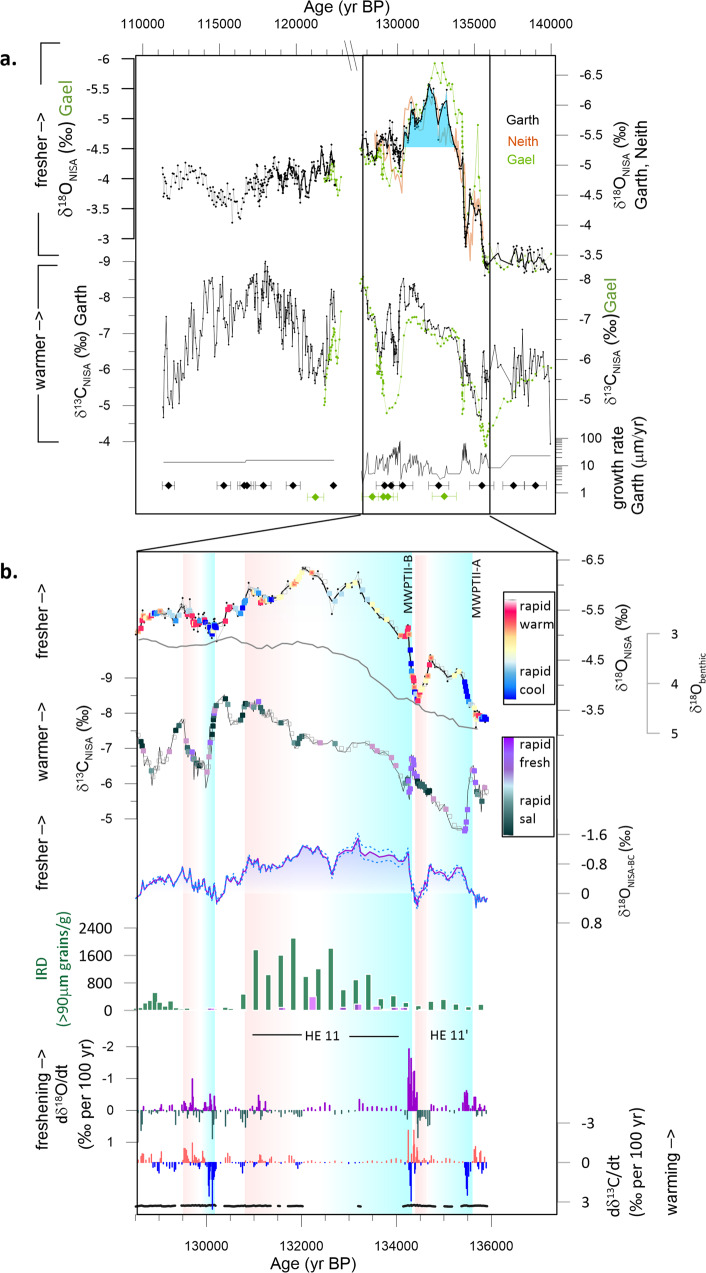
New NW Iberian speleothem records spanning TII. **a** Measured isotope records from stalagmites Garth (black), Neith (orange) and Gael (green), together with speleothem growth rate in Garth, and position of ^230^Th dates in Garth and Gael and their analytical error. A scale break is used for strongly condensed growth between 128 and 122 ka; full records in this time are shown in Supplementary Fig. 13 and 14. Blue infill in Garth record highlights when δ^18^O_NISA_ is >0.5 ‰ more depleted than at interglacial onset. **b** Detail of the deglacial period records from Garth, together with δ^18^O_benthic_ from MD01 _-_2444^59^ on speleothem age model as described in Supplement, and δ^18^O_NISA-BC_ (purple line with shading), the δ^18^O_NISA_ minus the benthic component of δ^18^O change, calculated as detailed in Methods, and the IRD abundance at ODP 983^109^ and MD01-2444^18^. The δ^18^O_NISA_ is color coded according to the rate of warming or cooling inferred from the rate of change in δ^13^C_NISA_ to highlight the temperature forcing and feedbacks on North Atlantic δ^18^O. Likewise_,_ the δ^13^C_NISA_ is color coded according to the rate of freshening inferred from the rate of change in δ^18^O_NISA_. Rate of change in δ^18^O_NISA_ and δ^13^C_NISA_ (_‰_) per 100 year, uses the smoothed isotope records from speleothem Garth shown. Bottom line indicates periods during which seasonally resolved fluorescent layers are present and used to define speleothem growth rate. Consistent with definition of HE by IRD, we designate the period initiating with MWP-TIIB as HE11, and denote the earlier phase of meltwater and AMOC reduction HE11’.

In the same coastal cave systems, we document the evolution of δ^18^O_NISA_ and δ^13^C_NISA_ over TII as indicators of δ^18^O_sw_ and temperature, respectively. Three ^230^Th–dated NISA stalagmites replicate the main features of the TII deglaciation between 135 and 129 ka (Fig. 4a). Annual countable fluorescent banding in stalagmite Garth (Supplementary Figs. 5 and 6) additionally provides a precise estimation of the rate of the main δ^18^O_NISA_ transitions. The record from stalagmite Garth extends to 112 ka BP and provides further context for the deglaciation, albeit with slowed growth and lower resolution between 127.5 and 122.5 ka. We assess the potential effect of in-cave processes such as PCP on the isotope records to focus on the most robust proxy trends (Methods, Supplementary Figs. 7–10) Given the correlation between δ^18^O_NISA_ and marine microfossil records of δ^18^O_sw_ from west and south Iberia in TI (Fig. 1), we tune marine sediment age models to speleothem chronology by synchronizing the major freshening associated with deglacial ice melting and some key temperature events (Methods, Supplementary Figs. 11–15).

δ^13^C_NISA_ records the stepped deglacial warming from 134 to 128 ka, punctuated by millennial cooling events, while δ^18^O_NISA_ records the deglacial freshening of the eastern North Atlantic beginning 135.7 ka (Fig. 4a). The subsequent descent into the last glacial cycle is also reflected in the increase in δ^18^O_NISA_ after 122 ka and persistent cooling in δ^13^C_NISA_ after 118 ka which suggest temperatures similar to the PGM by 112 ka. The annual layer counted chronology in many sections of stalagmite Garth resolves significant centennial to millennial scale variations in both the rate of eastern North Atlantic freshening (δ^18^O_NISA_) and its relationship with regional warming (δ^13^C_NISA_) (Fig. 4b).

Two large freshening pulses characterize the TII onset (MWPTII-A and MWPTII-B, Fig. 4b). Following previous studies^32^, we infer that the addition of meltwater is the main driver of rapid deglacial freshening and declines in the δ^18^O_sw_ in the eastern North Atlantic. Both abrupt freshening pulses began during a period of rapid warming, then, after several decades of rapid freshening, temperatures cooled rapidly, consistent with freshening-induced AMOC slowdown (Fig. 4b). Yet despite cooling, the freshening rate remained high for nearly a century during both events. Following the first period of rapid meltwater release (MWPTII-A), a local freshwater δ^18^O anomaly was maintained in the eastern North Atlantic for a duration of ~ 600 yrs before the rate of warming increased and over several centuries the surface ocean δ^18^O anomaly was reduced (Fig. 4b). We infer that slowed meltwater flux and re-invigorated AMOC dissipated the δ^18^O anomaly through dilution and mixing of meltwater throughout the global ocean.

**Fig. 5:**
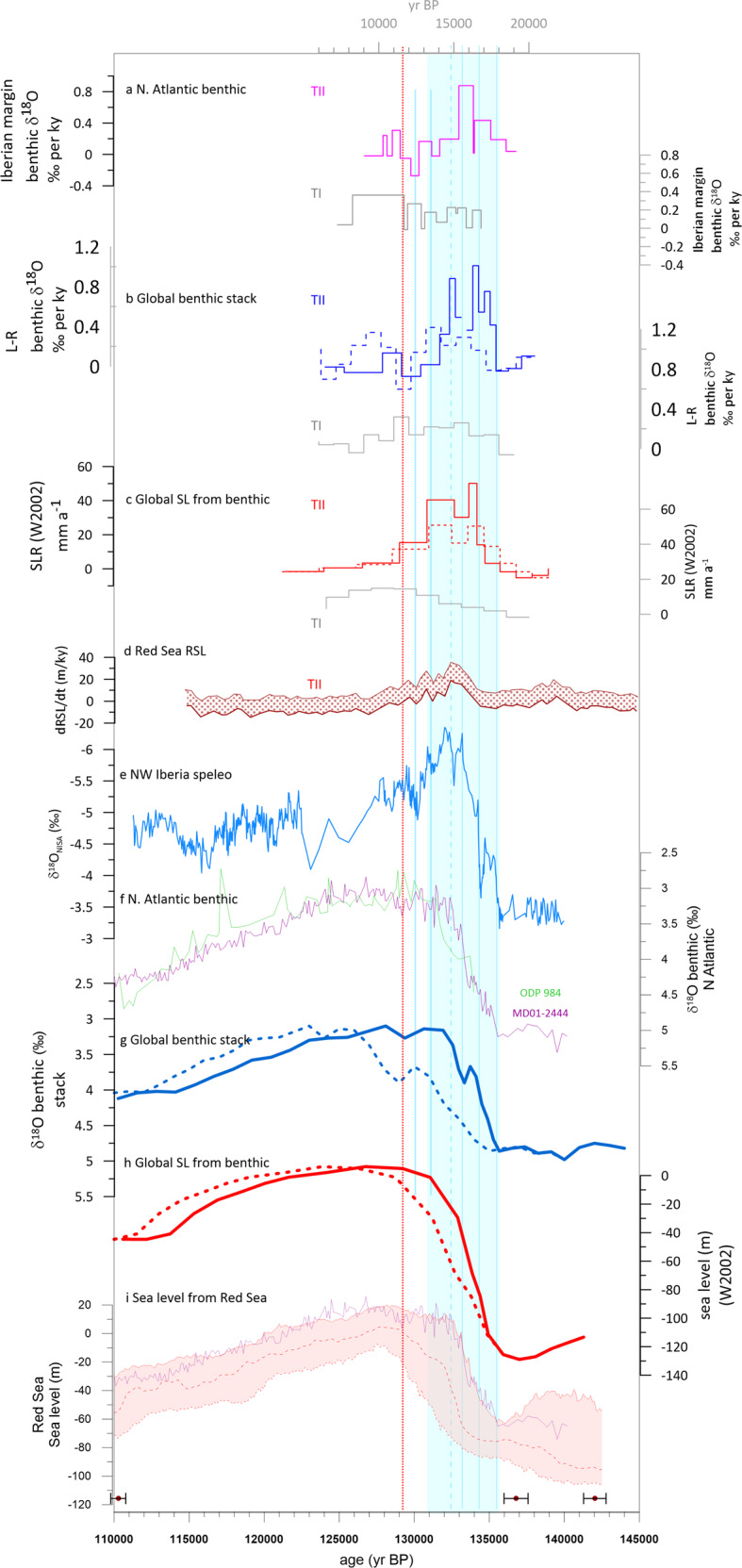
Rates of change in δ^18^O_benthic_ and sea level during TII. **a**–**d** Compare rates of change in δ^18^O_benthic_ and sea level records during TI and TII. The respective benthic δ^18^O_benthic_ and sea level curves spanning TII are illustrated in panels **f**–**i**. The NW Iberia δ^18^O_NISA_ from Garth is shown in **e**). Sources are: W Iberian margin δ^18^O_benthic_ records in TII^18,59^ following tuning to NW Iberian speleothem chronology (purple); comparison with rate of change in W Iberian margin δ^18^O_benthic_ records in TI^94,110^. Global δ^18^O_benthic_ stack^35^ for TII on original chronology (dashed red line) and our proposed maximum rate chronology (solid red line). Waelbroeck 2002 sea level curve from δ^18^O_benthic_ stack^37^ for TII on original chronology (dashed blue line) and our proposed maximum rate chronology (solid blue line). The Red Sea sea level record and its 95% CI is shown with red shading on its published chronology the 95% confidence interval estimated on the rate of sea level change is shown as reported^40^. At the base of the figure are shown the three age control tie points tuning the Red Sea record to a Mediterranean δ^18^O_planktic_ record (LC41) and their uncertainties^38^. These anchor the age model used to calculate the rate of freshwater addition from the derivative of the sea level curve. The uncertainties in the original Red Sea chronology do not preclude the rates of sea-level change we estimate through application of speleothem chronology to δ^18^O_benthic_. Blue vertical bar highlights HE11 as in Fig. 3, and the timing of NAIS outburst event is shown with red line. **i** The W. Iberian margin δ^18^O_benthic_ is shown overlain on the Red Sea Sea level curve to facilitate comparison of these two independent chronologies.

Following the second and most intense negative δ^18^O shift (MWPTII-B) and AMOC slowdown, a North Atlantic freshwater δ^18^O anomaly persisted. Unusually, for ~ 3000 years, the δ^18^O_NISA_ remained lower than the final interglacial state. The maintenance of a freshwater δ^18^O anomaly for so long in the surface eastern North Atlantic requires addition of meltwater at rates greater than it can be distributed through mixing with the global ocean. Thereafter, starting around 131 ka, the North Atlantic freshwater δ^18^O anomaly diminishes rapidly, which suggests that the rate of addition of meltwater into the eastern North Atlantic had slowed sufficiently so that ocean circulation homogenized the deglacial meltwater anomaly to the global ocean average. This decline coincides with a rapid decrease in IRD delivery in the Northeast Atlantic and Labrador Sea (Fig. 4b, Supplementary Fig. 12). The rate of decrease in δ^18^O of benthic foraminifera (δ^18^O_benthic_) on the Iberian margin also slowed significantly at 131 ka (Figs. 4 and 5), consistent with slowed melt rate.

We detect late phases of melting between 130 and 129 ka. A rapid negative shift in δ^18^O_NISA_ around 129.7 ka, during regional warming, likely reflects acceleration of meltwater addition. Notably, the continued meltwater flux through 129.3 ka appears to coincide with the deposition of a distinctive sediment layer on the Labrador margin^33^ which occurred at ~129.3 ka on our chronology (Supplementary Fig. 13), a depositional event attributed to a final North American Ice Sheet (NAIS) flood outburst, analogous to the 8.2 ka event following TI. The 8.2 ka event initiated when ~90% of North American ice had melted^34^, so if the sediment layer at 129.3 ka is analogous^33^, it suggests that a similarly high fraction of melting of PGM NAIS ice was likewise complete by this time.

On the W Iberian margin, our speleothem based chronology for TII indicates a much faster rate of depletion in δ^18^O_benthic_ during TII than during TI, suggesting that deep waters in the North Atlantic underwent freshening much more rapidly in TII than during TI (Fig. 5). The chronology of the global marine δ^18^O_benthic_ stack^35^, used as index of glacial ice volume to estimate rates of deglaciation and as inputs for model calculations^36^ is based on tuning to NH summer insolation, assuming a comparable phase relationship to TI. δ^18^O_benthic_ curves have also previously been used to estimate sea level^37^ and the rate of sea level rise^9^ during TII compared to TI. However, our chronology suggests that the rapid TII deglaciation precedes the peak in high latitude NH summer insolation and the existing chronology^35^ probably yields minimum estimates of rates of deglaciation. The speleothem chronology for the δ^18^O_benthic_ on the W Iberian margin provides an alternate estimate of the rate of sea level change and deglaciation (Fig. 5). This likely represents the upper estimate of the rate of sea level rise, duration, and amplitude of freshwater forcing compared to those employed in recent models using original chronology^9^. Our independent tuned chronology for δ^18^O_benthic_-based sea level estimates are closest to the oldest chronology (95% upper CI) for the Red Sea curve^38,39^, and our chronology implies the potential for an earlier and even more concentrated meltwater pulse than estimated from the derivative of the Red Sea sea level curve (Fig. 5)^40^.

### Similarities and contrasts between the meltwater anomaly during TI and TII deglaciation

**Fig. 6:**
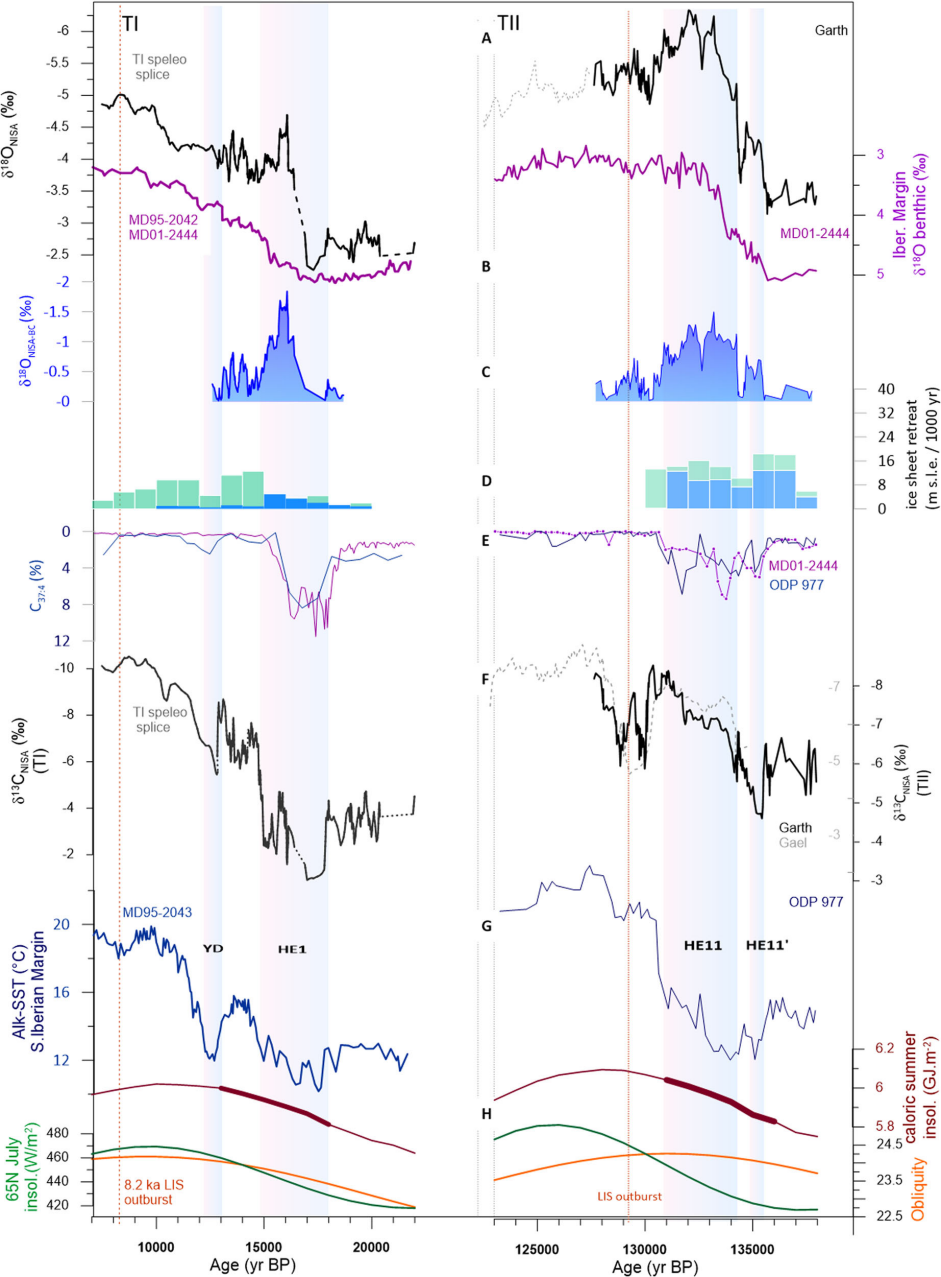
Comparison of deglacial evolution in North Atlantic during TI and TII. Synthesis of speleothem records from NW Iberia over TI (left panel) and TII (right panel) and marine records tuned to NW Iberian speleothem chronology. **A** Black line shows δ^18^O_NISA_ record from speleothem Garth (black line) and Gael (dashed line) for TII and TI speleo splice (see Supplementary Figs. 2 and 13). **B** Iberian Margin δ^18^O_benthic_^18^. **C** Eastern North Atlantic meltwater δ^18^O anomaly estimated from δ^18^O_NISA-BC_ calculated as described in Methods (**D**) rate of NH ice sheet melting in 1 ky age bins, for TI from^34,41^ and TII^8^. **E** C_37:4_ abundance on the W. and S. Iberian margin^58,59^ indicator of sea ice proximity (**F**) δ^13^C_NISA_ from Garth and TI splice, as temperature indicator (**G**) alkenone SST in the S. Iberian Margin^92,95^. **H** Variation in orbital parameters: caloric summer at 55°N (brown) obliquity (orange), and 65°N July insolation (green), with bold color highlighting caloric summer interval when eastern North Atlantic freshens faster than the global average. Vertical dashed red line indicates the NAIS outburst flood at end of TII^33^ and the 8.2 ka outburst linked to final NAIS collapse^111^.

Our speleothem absolute chronology confirms that both TI and TII deglaciations initiated at a similar threshold of caloric summer insolation (5.8 GJ m^−2^) (Fig. 6). Yet, the speleothem chronology for δ^18^O_benthic_ reveals attainment of interglacial values within ~5 ky of the TII deglacial onset, compared to nearly 9 ky in the TI deglaciation. Notably, the development of the eastern North Atlantic δ^18^O_sw_ anomaly during TII is markedly different from that of TI (Fig. 6). During TII, δ^18^O_sw_ remained more negative than the final interglacial state for nearly 3000 years, a situation which never occurred in TI. During TI, the regional surface ocean δ^18^O_sw_ anomaly, calculated relative to the benthic δ^18^O_calcite_ on the Iberian margin ((δ^18^O_NISA-BC_; Methods), remains below −1‰ only between 16.4 and 15.2 ka (1.2 ky duration), whereas during TII it remains below −1‰ from 134.7 to 131.2 (3.5 ky duration; Fig. 6).

**Fig. 7:**
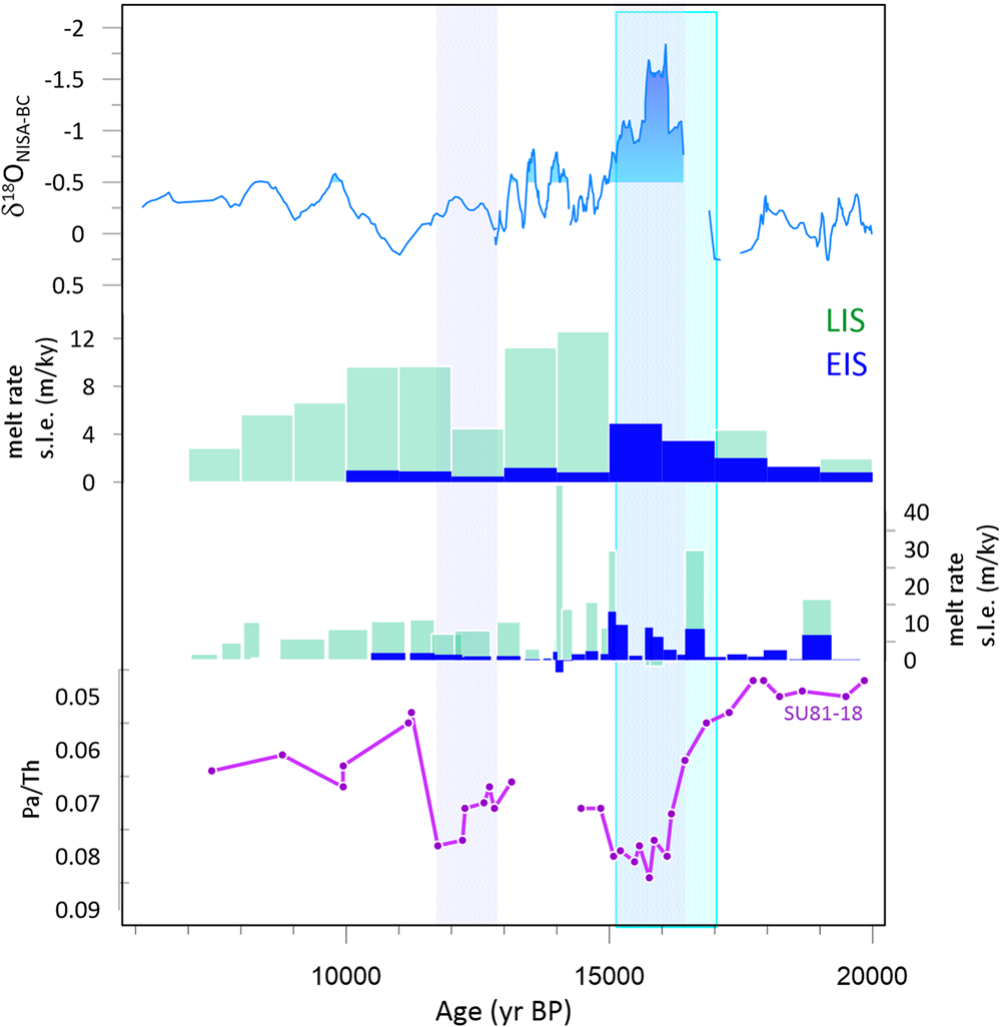
Time series of regional δ^18^O_sw_ anomaly over TI vs NH melting rate and AMOC indicators. Regional freshwater anomaly δ^18^O_NISA-BC_ compared with estimations of the melting rate NAIS and EIS, from^41^ (20 to 12 ka) and from^34^ (6–12 ka). Shown are melt rates derived from sea level curves interpolated to uniform 1 ky resolution, to facilitate comparison to TII available only at this resolution (Fig. 3), as well as rates at original published resolution. Also shown is the Pa/Th indicator of AMOC intensity^50^.

During TI, this period of greatest regional δ^18^O_sw_ anomaly coincides with the interval of fast melt rate of the Eurasian ice sheet according to recent sea level reconstructions^41^ (Fig. 7; Supplementary Fig. 16). One interpretation is that EIS meltwater leads to a greater negative δ^18^O_sw_ anomaly in the eastern north Atlantic compared to NAIS meltwater. Southward advection of surface waters from the Nordic seas along the European continental margin is simulated during periods of retreat of the northern and western EIS^20^. Intermediate complexity models comparing identical freshwater forcing in different outlets infer a greater salinity anomaly in the eastern North Atlantic due to Eurasian meltwater compared to North American meltwater routed through Hudson Bay, Gulf of Mexico, or Gulf of St. Lawrence. This is because for these NAIS routes, the salinity (and δ^18^O_sw_ anomaly) would be diluted during advection across the Atlantic by the North Atlantic Drift^42^. These model simulations, albeit limited, suggest the potential for higher amplitude δ^18^O_sw_ anomaly in the eastern North Atlantic during EIS melt than NAIS melt, given similar δ^18^O_ice_ as assumed in previous studies^43^. Therefore, one explanation for the longer duration surface ocean δ^18^O_sw_ anomaly during TII is a larger proportion of EIS-derived meltwater in TII compared to TI.

In support of this explanation, Eurasian PGM deposits are found well beyond the limit of ice extent from the LGM, with the largest expansion in the Barents-Kara sectors of northern Russia (Fig. 1). However, the sparse direct chronology of ice positions does not distinguish if these advances were synchronous across all sectors to yield at a single time during the penultimate glacial a significantly larger EIS, or if, as in the last glacial cycle, sectors were out of phase^44,45^. The potential for a larger PGM EIS ice sheet has been explored in dynamical ice sheet models with total EIS ice volumes of up to 70 m sea-level equivalent, nearly triple that of the LGM EIS^46^. Because of similar estimated PGM and LGM sea level and benthic δ^18^O, this simulated large PGM EIS likely coexisted with a smaller than LGM NAIS^46^. The ice sheet reconstruction featuring a large TII EIS^46^ encompasses a 2.5–3.5x greater volume of marine-grounded (grounded below paleo sea level) EIS ice at the PGM, compared to the LGM (Methods) with thickest ice in the Fennoscandian sector. Because of isostatic loading and higher relative sea level caused by self-gravitation, the larger PGM EIS would have been even more sensitive to ocean forcing and marine ice sheet instability which could have contributed to a rapid retreat. Although the onset of modeled TII EIS retreat differs slightly from our record^8^, its duration is broadly consistent with the here documented duration of eastern North Atlantic δ^18^O anomaly.

An alternative explanation for the greater freshening in the eastern N Atlantic during TII is that LGM-like ice sheet configuration melted faster in TII than in TI. However, we consider this scenario less likely because it would require faster melting of the portions of NAIS which were longest lived during TI, and we have no evidence of a stronger forcing during TII. An Arctic ice shelf, neutrally buoyant but potentially containing a freshwater equivalent to 10 m s.l.e. is proposed to have existed at some time during the penultimate glacial cycle but the timing of formation and collapse remains controversial^47–49^. Based on our records, we cannot confirm nor rule out disintegration of such an ice shelf as a contributor to freshening at the onset of TII, leading the retreat of Northern sectors of the EIS. However, the modest magnitude of Arctic shelf ice volume suggests that this cannot be the only factor responsible for differences in the meltwater anomaly and rate of change in δ^18^O_benthic_ and sea level rise over TII (Figs. 5 and 6).

In addition, models suggest that the intensity and duration of the regional δ^18^O_sw_ meltwater anomaly are also increased by a slowing of the rate of dilution of the meltwater signal, primarily because slowed overturning circulation reduces the vertical dissipation rate^11^. During TI, the greatest regional freshening anomaly (16.4 to 15.2 ka) also coincides with a period of AMOC reduction inferred from proxies such as Pa/Th^50^ (Fig. 7, Supplementary Fig. 16). For this time interval, the relative importance of the EIS meltwater source vs AMOC amplification of meltwater anomaly cannot yet be directly deconvolved because existing Pa/Th records suggest only a 30% AMOC reduction during HE1^51^ while the high resolution isotope-enabled OA-GCM experiments simulate a very extreme AMOC shutdown (from 16 to 2.5 Sv). Nonetheless, the temporal coincidence of reduced AMOC and the phase of most rapid EIS retreat in TI may not be coincidence. Simulations suggest that meltwater release from the Northern and western margin of the EIS (and the Mackenzie River outlet of the North Americans), is more effective at decreasing AMOC compared to North American melt routed through the Gulf of Mexico, Hudson Bay, or St. Lawrence outflows^42,52^, because the former meltwaters reach the locations of northern North Atlantic convection with lesser dilution by other surface waters^53^. Therefore, the strong surface meltwater δ^18^O_sw_ between 16.4 and 15.2 ka in the eastern north Atlantic may be both a direct consequence of high EIS melt rates releasing water in the eastern North Atlantic, and an indirect consequence of the high EIS melt rates on AMOC stability and concomitant amplification of the surface meltwater δ^18^O_sw_. In and of itself, reduced AMOC intensity, such as that inferred to have occurred during the Younger Dryas according to Pa/Th (Fig. 7, Supplementary Fig. 16), does not coincide with a large freshwater δ^18^O_sw_ anomaly in the eastern North Atlantic. This suggests that the EIS source effect may be the dominant factor leading to the surface meltwater δ^18^O_sw_ anomaly early in TI. This hypothesis should be further explored in experiments in high resolution coupled OA-GCM simulations with meltwater release in realistic regions, also exploring the effect of a range of AMOC reduction intensities on the rate of dissipation and therefore the amplitude and persistence of meltwater δ^18^O_sw_ anomaly.

Further evidence for continual meltwater flux during the long TII meltwater anomaly comes from marine IRD records. On our speleothem chronology, marine records show that peak IRD deposition in both the northeastern Atlantic ODP sites 983 and 984 as well as in sites around the Iberian Margin coincides with the long δ^18^O_NISA_ anomaly. Because large IRD fluxes are commonly interpreted to reflect a high flux of ice calving from instability in marine-terminating ice sheets^54^, the IRD peak suggests that the long surface ocean δ^18^O anomaly was not the result of slowed ocean circulation alone, but that continued meltwater addition from decaying ice sheets either contributed to or was the dominant driver of the signal (Fig. 4b). Large North Atlantic IRD and Heinrich Events (HE) have been linked to the large meltwater release during multiple glacial terminations^55^. We suggest this period corresponds to HE11, and distinguish the first freshening-induced AMOC slowdown of MWPTII-A as HE11’. While the amplitude and duration of the eastern North Atlantic δ^18^O anomaly may potentially be enhanced if the release of meltwater shuts down AMOC^11^, we consider it unlikely that a more intense or longer duration of AMOC weakening during TII is the only cause for the long period of extreme eastern North Atlantic δ^18^O fresh anomaly. Although there are no direct quantitative proxies for AMOC intensity in TI or TII^51^, some mid-latitude proxy records are likely to be sensitive to the strong winter season climatic impacts of AMOC slowdown such as the relative abundance of *Neogloboquadrina pachyderma sinistral*^56^ or relative abundance of C_37:4_ alkenone^57–59^ (Fig. 6). These climatic indicators suggest a less extreme AMOC slowdown and sea ice response to stratification during HE11 in TII than in HE1 during TI (Fig. 6, Supplementary Fig. 9). The duration of HE11 and HE1 are comparable. Thus, available evidence would not support a difference in physical circulation as the only cause for the contrasting surface ocean eastern North Atlantic δ^18^O anomaly in TI vs TII.

Based on these considerations, we suggest that our record of freshening is most consistent with a PGM EIS much larger than its LGM counterpart, and is compatible with the larger EIS volume scenarios of 60 m to 71 m sea level equivalent^60,61^ based on glacial-isostatic modelling. Further ice sheet modeling and assimilation of near field geophysical data are required to evaluate this interpretation.

### Causes and feedbacks from meltwater-induced AMOC disruption

Our new, highly resolved record of TII reveals that, as in TI, the deglacial sequence was characterized by a series of millennial-scale variations not previously resolved in records of sea level rise^35,37–40^. We find evidence for multiple phases of AMOC reduction and reinvigoration in TII which provide new clues to the mechanisms of AMOC instability during glacial terminations. It has been debated, whether meltwater release causes AMOC reduction, or if it is the subsurface ocean warming during AMOC shutdown that causes accelerated ice sheet collapse and meltwater release^53^. Here, our new annually laminated records show that enhanced freshwater addition during brief periods of rapid warming led, and therefore likely caused, AMOC reductions early in TII.

AMOC recovery has most often been attributed to a slowing of the rate of meltwater addition. Alternatively, recent model experiments suggest that AMOC can also recover, despite sustained meltwater flux, if there is a change in the boundary conditions which set the threshold for weakened AMOC. For example, AMOC may have recovered after HE1 in TI during the Bølling-Allerød because the rapid CO_2_ rise during HE1 raised the forcing threshold required to maintain weak AMOC^5,62^. TII was previously proposed to lack such a mid-termination AMOC recovery^5^. Yet, we identify a recovery after the first AMOC reduction of TII (Fig. 4b). This event occurred earlier in the deglaciation (e.g. δ^18^O_benthic_ on the Iberian margin was 4.45 ‰ during the TII recovery compared to 4.25‰ at the start of the Bølling-Allerød) (Fig. 6). One possibility is that AMOC recoveries at TII may have been driven not by evolving thresholds in AMOC sensitivity but rather by strong temporal variations in meltwater forcing. This short duration of the first TII AMOC reduction (600 years vs ~3000 years for HE1 in TI) may reflect the operation of a negative feedback of AMOC slowdown on TII melting rate, if the main location of early TII melting occurred in an area sensitive to AMOC-induced cooling.

**Fig. 8:**
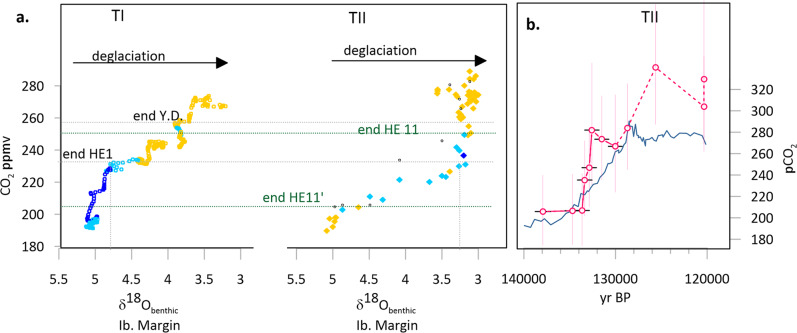
Evolution of atmospheric pCO_2_ during TI and TII. **a** The evolution of ice core pCO_2_ for TI^64^ and TII^7^ versus benthic δ^18^O on the Iberian margin; benthic δ^18^O tuned to speleothem chronology for TII. Symbols are colored according to the C_37:4_ alkenone abundance in the S. Iberian margin^58^ (dark blue C37:4 > 6%; light blue C37:4 2–6%; yellow <2%) as an indicator of timing and intensity of HE climate impact. Horizontal gray lines emphasize CO^2^ levels reached following abrupt rises during stadials in TI (HE1 and Y.D.), and horizontal dark green lines highlight CO_2_ levels attained at the end of stadials in TII (HE11’ and HE11). **b** Comparison of the TII temporal evolution of CO_2_ on ice core chronology (gray curve)^7^ with pCO_2_ estimated from marine proxies (pink, vertical bars show 95% CI on CO_2_ estimate)^66^ on speleothem chronology as discussed in Methods (horizontal bars show + /−750 year uncertainty on age). The TII marine proxy pCO_2_ estimates from between 138 and 128 ka are shown with small black squares in a), with error bars omitted for clarity.

On the other hand, if a change in boundary conditions is required for AMOC recovery, then our results in TII present a paradox, because according to the current available ice core chronology, the onset of the first AMOC recovery occurred when CO_2_ had risen only to 207 ppmv^7^, much less than the 245 ppmv attained by the onset of the Bølling-Allerød (Fig. 8)^7^. Even by the end of HE11, according to AICC2012 chronology, pCO_2_ had only reached 245 ppmv, much less than the 260 ppm reached by the end of the Younger Dryas. This ice core CO_2_ chronology would suggest a very different sensitivity of AMOC to CO_2_ between TI and TII. This circumstance could reflect other initial boundary conditions, such as a lower glacial height of the North American ice sheet or different initial PGM thermohaline regime^63^, which conditioned the AMOC threshold.

It is also possible that ice core records may underestimate the rate of TII CO_2_ rise due to the differing precision of age models based on annual layer counting for TII^64^, and age models based on age interpolation between two age control points at 121 and 135 ka tuning ice core O_2_/N_2_ ratios to local summer insolation^65^. The chronology of the main TII CO_2_ rise can be further explored from an available marine CO_2_ proxy record based on planktic foraminifera δ^11^B from the western Caribbean^66^. This record indicates a large initial CO_2_ rise synchronous with a surface ocean freshening recorded by the same planktic foraminifera^67^. Although tuning this site to our speleothem chronology has some uncertainty (Methods, Supplementary Fig. 17), and proxy CO_2_ estimates are less precise than direct measurements in ice cores, a conservative correlation suggests that a larger portion of the deglacial CO_2_ rise may have occurred rapidly, coincident with the onset of rapid EIS melting and the second AMOC slowdown (Fig. 7). An early and rapid CO_2_ rise, introducing greenhouse-forced warming, is also consistent with the progressive warming above glacial background levels occurring after MWP-TIIB in our air temperature record, which would have maintained the melting of NH ice sheets during the HE11 AMOC slowdown (Fig. 8). Thus, diagnosing the links between rate of ice melting, AMOC recovery, and deep ocean carbon storage during TII may require reassessment of the rate of CO_2_ rise.

Overall, the first direct absolute age constraints on the timing and rate of TII freshwater release to the North Atlantic are consistent with a large EIS, which was prone to rapid retreat due to its enlarged marine-based boundaries. These observations highlight the control that glacial boundary conditions exert, by shaping ice sheet anatomy, over the rates of ice melting and also the nature of the subsequent interglacial^9,68^. The new records also provide the first evidence for discrete centennial scale meltwater pulses indicative of phases of accelerated ice sheet failure during TII and a detailed view of their coupling with cooling consistent with AMOC reductions. The role of internal ice processes and climate feedbacks associated with the retreat of large marine-based northern hemisphere ice sheets should be further investigated with coupled ocean-climate-ice sheet models.

## Methods

### Identification of final moisture source exchange via backtracking

Moisture uptake regions of 104 rain events which were sampled <100 km west of the cave during 2015 and 2016, were calculated using backward-trajectory analysis with 1 h temporal resolution, performed using the Hybrid Single-Particle Lagrangian Integrated Trajectory (HYSPLIT) Model (Version 4.8)^69^ and following a similar methodology as previous studies^70^. Moisture uptake regions along 850 hPa, 700 hPa and 500 hPa were identified using a specific humidity threshold of 0.5 g/kg increment in at least 6 h, using meteorological data provided by backward-trajectories. Results are shown in Fig. 2d.

### Stalagmite samples

Stalagmite samples were analyzed from the following previously described^71^ NW Iberian caves: Pindal Cave (stalagmites Candela, Laura), La Vallina Cave (Garth, Gael, Galia, Gloria), and Cueva Rosa (Neith, Alicia). These stalagmites cover TI, Greenland Stadial 22 (GS22), and TII^27,71,72^. Additional growth phases have been studied in the stalagmite Candela since earlier publication^72^, and we report additional dates and higher resolution geochemical data. In Candela, A ~10 cm long basal growth phase (CANB) has been sampled along its main growth axis and redated, contrasting with strongly off-axis and condensed sampling of this phase in earlier work. Additionally, within the Bølling-Allerød, a transient 1.5 cm shift in growth axis has been resampled along its expanded central growth axis for 2.5 cm, expanding by 5-fold the record previously sampled in a lateral portion at only 0.5 cm thickness.

### Measurement of seasonal fluorescent banding for growth rate evaluation in key intervals

To account for variation in growth rates^73^ between ^230^Th dates, for intervals of rapid freshening in TI and TII, we refine age models by estimating stalagmite growth rates from the width of growth bands defined by fluorescent layers^74^ which have been described to result from seasonal delivery and/or incorporation of fluorescent organic substances in dripwater^75^.

Stalagmite slabs were mounted in low-viscosity Laromin C260 epoxy (cycloaliphatic diamine polymer), ground flat with SiC paper, and then polished with a diamond suspension to 0.25 µm in preparation for Confocal laser scanning microscopy (CLSM) and laser ablation ICP-MS. Imaging by CLSM was performed at the Scientific Center for Optical and Electron Microscopy (ScopeM) at ETH Hönggerberg using an OIympus Fluoview 3000. Here, a series of overlapping images were obtained under 100–200x magnification using an incident wavelength of 488 nm and measuring fluorescence in a 490–590 nm window. Images were obtained at 1024 × 1024 or 2048 × 2048 resolution, corresponding to 1.2 to 0.6 µm/pixel (respectively) under 100x magnification. Images were processed using Fiji/ImageJ, where the exact distances between growth layers were recorded on an absolute length scale and later cross-referenced to laser ablation and drilling (isotope, trace element) data. Confocal images were counted in stalagmite Garth section from 177.5 to 269.5 mm from the tip. Growth layer thickness was used to determine rates of isotopic change in sections which could be layer counted, and to develop accurate interpolation of ages between U/Th dates. In areas where annual layers were not immediately visible, typically when layer thickness was less than 3 µm, an average growth rate for that section was assigned in order to be consistent with the sequence of U/Th ages.

### ^230^Th and ^14^C absolute dating and age model development

Absolute chronology is based on subsamples drilled for ^230^Th dating. New dating was completed using standard methods and analysis on MC-ICP-MS (Thermo-Finnigan Neptune)^76^ at Xi’an Jiaotong University and the University of Minnesota; some previously published aged used in this study also include determinations on SF-ICP-MS (Thermo-Finnigan ELEMENT^77^); results are given in Supplement Tables 4 and 6. To evaluate growth rate in stalagmite Laura, ^14^C ages were measured at ETH Zurich with a gas ion source in a Mini Carbon Dating System at the Laboratory of Ion Beam Physics, ETH, via on-line acid digestion^78^ and calibrated with INTCAL 2020^79^ and results are given in Supplement Table 5. No new age models are elaborated for GS22 and these are shown in^27^. Because agricultural and pasture land uses became widespread in Northern Spain after 5.5 ka BP^80^ which may decouple the natural relationships between climate and vegetation over cave sites, we do not present or discuss speleothem records younger than 5 ka BP.

Age models for stalagmites younger than 30 ky are illustrated in Supplementary Fig. 1. Sequences of multiple ^230^Th ages are interpolated using the Bchron software^81,82^ (Maria, Candela, Galia, and Alicia A) whereas linear interpolation was employed for short growth phases bracketed only by basal and top U/Th dates (Alicia B, C). Two approaches are used to refine the age model within the 18 to 14 ka interval in Candela. Where visible growth laminae are present, their thickness is used to constrain growth rates (green line in growth rate curve in Supplementary Fig. 1a) and to identify the point at which growth becomes most strongly condensed. For example, the age model is pinned at a new MC age at 18.0 ka ± 78 year ^230^Th date (at reference level 4.5 mm), and advanced upwards ~90 year using width of fluorescent growth layers to constrain growth rate. Growth layers are not countable in the following 7.7 mm of stalagmite (brown line in Supplementary Fig. 1a). Consequently, in this section two tie points with the geochemical variations in stalagmite Laura (red points in Supplementary Fig. 1a) were used to constrain the Candela age model, and growth rate was varied from 10 to 1 μm/year inversely with Candela δ^13^C, with a 630 year negligible deposition (<0.2 μm/year) interval at the end of the δ^13^C maximum. The precision of the age model is lowest between 17.85 and 16.1 ka, and improves by 16 ka due to a 15.4 ka ^230^Th date and the 16.1 and 14.5 ka tie points with Laura. Following these tie points, the age model until the hiatus at 11.6 ka is established from ^230^Th tie points, with interpolation between dated points further constrained by counting of 600 annual layers (14.5 to 13.9 ka) during the Bølling-Allerød. This set of speleothem records chronicles the changes across TI in higher detail than previous records from the region, with the following average absolute 95% CI age uncertainties 20–18 ka (Candela, +/−300 years); 18–16 ka (Candela, +/−500 years), 16–14 ka (Laura, +/−150 years); 14–12.5 ka (Candela +/− 260 years).

Age models for stalagmites spanning TII and the Bchron uncertainty windows, are illustrated in Supplementary Figs. 5 and 6. For TII, Garth provides anchor chronology due to its high U content and growth rate and presence of annual fluorescent growth banding; Gael also provides an independent chronology albeit of lesser resolution. For Neith, whose very low U content complicates a precise age model, we develop an age model by tuning main features of isotope record to that of Garth within the uncertainties of the Bchron model. Neith is therefore presented to indicate the reproducibility of the main features of the isotopic records shown in Garth, not as independent verification of precise event chronology. The age model in Garth was developed with the aid of laminae counting (Supplementary Fig. 5).

### Stable isotope analysis and TI stable isotope splice

New stable isotope determinations were measured at Scientific and Technologic Centers from the University of Barcelona with a Thermo-Finnegan MAT-252 coupled to a CarboKiel-II and at ETH Zürich with a Thermo-Finnegan Delta V Plus coupled to Gas Bench II^83^ and are reported in ‰ relative to the Vienna PDB Standard. Analytical uncertainties for calibration with two in-house carbonate standards and NBS-19 and NBS-18 international standard and were 0.08 ‰ for both isotopes. Samples for isotopes were micromilled at 0.1 to 0.5 mm increments, or drilled at 1 to 5 mm increments, from the central axis of stalagmites. Stalagmite δ^18^O and δ^13^C were screened for the coupled effects of CO_2_ degassing and prior calcite precipitation, as detailed below. A spliced record of δ^18^O_NISA_ and δ^13^C_NISA_ is compiled from stalagmites spanning 26–5 ka BP. Where multiple stalagmites grow synchronously, we employ for the splice, the stalagmite with best constrained chronology and the stalagmite in which there is the least variation in Mg/Ca_index_ over the included time interval. The goal of this approach is to limit the potential influence of PCP on the inferred trends in δ^18^O_NISA_ and δ^13^C_NISA_.

### Speleothem Mg/Ca analysis, Mg/Ca as PCP index, and screening for PCP influence on isotope records

Mg/Ca was measured on splits of the isotope samples, at the University of Oviedo (Thermo ICAP DUO 6300)^72^, and with similar standardization approaches at ETH Zürich (Agilent QQQ 8800); all ratios are reported in mmol/mol. Mg/Ca is widely used indicator of variation in PCP^84^ and we use speleothem Mg/Ca and the Mg/Ca_index_ as a qualitative indicator of the influence of the coupled process of CO_2_ degassing and prior calcite precipitation (PCP) on isotope trends, hereafter referred to as PCP. To facilitate comparison across stalagmites from different portions of the cave, which sample stratigraphically distinct portions of the host limestone, we normalize the measured Mg/Ca in each sample (Mg/Ca_sample_) to the minimum Mg/Ca of that stalagmite (Mg/Ca_min_), where high index suggests little loss of Ca due to PCP and a low index suggests significant loss of Ca due to PCP.

1 $${{{{{\rm{Mg}}}}}}/{{{{{{\rm{Ca}}}}}}}_{{{{{{\rm{index}}}}}}}=({{{{{\rm{Mg}}}}}}/{{{{{{\rm{Ca}}}}}}}_{{{\min }}})/({{{{{\rm{Mg}}}}}}/{{{{{{\rm{Ca}}}}}}}_{{{{{{\rm{sample}}}}}}})$$

Since the δ^13^C of dripwater acquired through rain equilibration with soil gas and bedrock dissolution is the primary signal of interest, we plot stalagmite δ^13^C coded by the Mg/Ca_index_ (Supplementary Figs. 4, 8, 10) to identify when there may be an additional component of variation in δ^13^C_NISA_ due to in-cave modification of dripwater δ^13^C due to CO_2_ degassing and PCP. Monitoring suggests that secondary factors such as changes in Mg partitioning, non-bedrock Mg sources, and variations in the congruency of bedrock dissolution due to variable water-rock contact times exert a limited influence on speleothem Mg/Ca in most of our studied stalagmites. We refrain from use of the Mg/Ca index, and interpretation of isotope results, for the <9 ka portions of Pindal Cave stalagmites in which the cave position on the sea cliff generates a strong marine aerosol Mg/Ca influence. Based on our finding that in stalagmites spanning TI, the main glacial/interglacial δ^13^C_NISA_ contrasts and main transitions do not coincide with unidirectional changes in Mg/Ca_index_ we conclude these are not artefacts of varying PCP.

In TII, the Mg/Ca_index_ is used to ascertain trends which are not influenced by PCP. In stalagmite Garth, the principal transition to strongly negative δ^13^C_NISA_136–130 ka occurs within a very narrow range in Mg/Ca (4.7 ± 0.37 (1 s) mmol/mol) (Supplementary Figs. 8, 10), confirming this δ^13^C_NISA_transition is not driven by variable PCP. However, Mg/Ca in Garth rises abruptly after 129 ka and between 127 and 122 ka, the coincidence of maximum Mg/Ca ratios and strongly condensed growth indicated by ^230^Th ages is most likely explained by enhanced PCP (Supplementary Fig. 10). Between 127 and 122 ka, δ^13^C_NISA_ may be shifted to more positive values than would be attained if PCP values were comparable to other sections of the stalagmite and therefore, PCP may be attenuating the magnitude of change in δ^13^C_NISA_ during peak MIS5e warmth in this stalagmite. Consequently, we rely on other stalagmite record for this interval. Stalagmite Gael, shows overall more constant Mg/Ca_index_ than Garth (Supplementary Fig. 8h), suggesting that relative trends in δ^13^C_NISA_ between early termination (134–130 ka) and peak MIS 5e (127–123 ka) are less influenced by PCP in Gael. At the same time, the trend in δ^13^C_NISA_ at the termination onset may be amplified in Gael by decrease in PCP between 135 and 134 ka. In stalagmite Neith, Mg/Ca and δ^13^C_NISA_ correlate strongly within glacial and termination time windows (Supplementary Fig. 8i) and although a shift in mean δ^13^C_NISA_ between glacial and interglacial is evident, PCP may be the dominant source of temporal variation in δ^13^C_NISA_ within each of these time windows, so we abstain from interpreting the time series of δ^13^C_NISA_ in Neith. We propose stalagmite Garth, with high and relatively stable Mg/Ca_index_ and most precise chronology, to present the most reliable δ^13^C_NISA_ record, except during its condensed interval between 128 and 122 ka; for this latter period, we splice in the temporal variation observed in stalagmite Gael so that long term trends can be accurately assessed.

We also use the Mg/Ca index to evaluate the potential effect of varying PCP on δ^18^O, since stalagmite δ^18^O may be isotopically enriched when forming from dripwaters which have experienced significant PCP at rates exceeding those of oxygen isotope equilibration between dissolved inorganic carbon and water, as observed in laboratory studies^85,86^. The magnitude of this effect in cave settings could be buffered by the large water reservoir exchanging with DIC especially if carbonic anhydrase from soil and cave microbial^87,88^ communities catalyzes exchange. Using previously published δ^18^O_NISA_ records from 100 to 80 ka^27^, we find that a lower Mg/Ca_index_ coincides with stalagmite sections offset to higher δ^18^O_NISA_ relative to coeval stalagmites, suggesting this index is useful to identify PCP-related influences on δ^18^O_NISA_ in these cave settings (Supplementary Fig. 7).

In stalagmites spanning TI, the major temporal trends in our δ^18^O_NISA_ records do not correlate with Mg/Ca_index_ in the intervals of each record selected for the splice (Supplementary Fig. 2). In the spliced record overall, there is no systematic correlation between Mg/Ca_index_ and the decrease in δ^18^O. Therefore there is no evidence the δ^18^O trends are artefacts of temporally variable PCP.

During TII, we evaluate whether deglacial δ^18^O_NISA_ changes coincide with changes in cave-influences (such as PCP) using crossplots of isotopic records with Mg/Ca (Supplementary Fig. 8). We color code data by age. This analysis confirms that in Garth, there is no correlation between Mg/Ca and δ^18^O_NISA_; the full transition of δ^18^O_NISA_from 136 to 132 ka occurs when the speleothem maintains a very narrow range in Mg/Ca (4.8 ± 0.35 (1 s) mmol/mol), suggesting that variation in PCP is not a significant influence on the trend in δ^18^O_NISA_ in this time interval. Over the termination there is also no correlation between growth rate and δ^18^O_NISA_ in Garth, the stalagmite for which growth rate is most precisely resolved due to annual layer counting. (Supplementary Fig. 8). Although the timing and features of the δ^18^O_NISA_ are similar in stalagmite Gael, its δ^18^O_NISA_ is on average 1% higher compared to stalagmite Garth or Neith. Gael has a lower average Mg/Ca_index_ than the other two stalagmites, suggestive of greater PCP which could lead to offset of the absolute δ^18^O_NISA_ for stalagmite Gael. Additionally in Gael, the shift to slightly lower mean value of Mg/Ca midway through the termination onset coincides with a reduced offset in δ^18^O_NISA_ between Gael and Garth (Supplementary Fig. 9), amplifying the δ^18^O_NISA_ transition in Gael between 135 and 134 ka by about 0.5%. In Neith, there is no correlation between δ^18^O_NISA_ and Mg/Ca, and for Gael and Neith, growth rates are less precisely constrained but there is no consistent correlation between average growth rate and δ^18^O_NISA_. Therefore, we employ stalagmite Garth, with high and relatively stable Mg/Ca_index_ and most precise chronology, as our core δ^18^O_NISA_ record, except during its condensed interval between 128 and 122 ka, when the period of most significant PCP may increase the δ^18^O_NISA_ compared to other time intervals in that stalagmite. During the interval of condensed growth in Garth, we splice in the temporal variation observed in stalagmite Gael, plotting them on a separate y-axis so that long term trends can be accurately assessed. We employ the record from stalagmite Garth to estimate the eastern North Atlantic surface ocean δ^18^O_sw_ anomaly relative to the Iberian margin δ^18^O_benthic_ (δ^18^O_NISA-BC_; Methods).

### Estimation of δ^18^O_sw_ relationship with δ^18^O_NISA_ over TI and calculation of surface ocean anomaly

The δ^18^O of surface seawater (δ^18^O_sw_) is commonly estimated from coupled Mg/Ca and δ^18^O of planktic foraminifera^89^. We compare published marine δ^18^O_sw_ with δ^18^O_NISA_ over the time interval from 25 to 5 ka BP in fixed 500 year time bins (Fig. 2, Supplementary Fig. 3). The marine records are based on original ^14^C chronology which for the S Iberian margin assumes constant reservoir age^17^. For the W Iberian margin we apply recent age model assuming variable reservoir age^90^. For the Irish margin, the original chronology is based on tuning the % *N. pachyderma sinistral* relative abundance to the GISPII δ^18^O variation since ^14^C dates suggested variable reservoir age^32^. Superimposed multicentennial- scale variability is evident in both δ^18^O_sw_ and δ^18^O_NISA_, but at this time cannot be confidently independently correlated due to the uncertainty of marine ^14^C-based and correlation-based age models.

The calculation of regional surface ocean δ^18^O_sw_ anomaly as a surface ocean δ^18^O_sw_ minus a global δ^18^O “ice volume effect” is subject to uncertainties in the age correlation between the δ^18^O_sw_ and the eustatic sea level curve used to calculate the ice volume effect. To reduce this source of uncertainty and to use a similar metric for TI and TII, we instead describe the local surface ocean δ^18^O_sw_ (δ^18^O_NISA_) relative to the benthic δ^18^O_calcite_ on the Iberian margin, which can be precisely and independently tuned to speleothem chronology for TII. This metric is denominated (δ^18^O_NISA-BC_) or (δ^18^O_SW-BC_). Although the decrease in benthic δ^18^O_calcite_ is driven by both deep ocean warming and decreasing deep ocean water δ^18^O components, high resolution OA GCM model simulations show that in the depth range of our Iberian Margin North Atlantic records, decreasing deep ocean δ^18^O and warming are closely temporally coupled when meltwater release decreases AMOC intensity, because shallowed North Atlantic winter mixed layer depth lead to mid-depth warming^11^.

### Tuning of marine age models for Iberian Margin to speleothem chronology

We compile published δ^18^O_sw_ records for TII^18,91^ based on Mg/Ca SST and δ^18^O_calcite_ and align the major depletion in both δ^18^O_sw_ of ODP 977^91^ and in δ^18^O_NISA_ in Garth, as illustrated in Supplementary Fig. 11. Because NW Iberian caves also lie in the center of a broad region affected by pronounced cooling during AMOC weakening events^10^, we extend the NW Iberian speleothem chronology by the additional alignment of three key cooling events in the δ^13^C_NISA_ record with events identified in the alkenone-SST record also from ODP 977^92^. ODP 977 is the primary tuning target because is the only available δ^18^O_sw_ record that matches exactly the amplitude of the initial speleothem freshening in both TI and TII (Fig. 6; Supplementary Fig. 13), likely because by the strong density gradient between the surface melt waters and the underlying Mediterranean waters maintains strong surface stratification, but also because higher sedimentation rates in ODP 977 may better preserve the original δ^18^O_sw_ amplitude (Supplementary Fig. 12). The tuning is based on tie points between 135 and 125 ka leading to highest precision in the marine chronology during this tuned interval and we do not discuss the details of marine records prior to MWPTII-B.

The ODP 977 marine chronology is transferred to ODP 976^93^ on the S Iberian margin and MD01-2444^18,59^ in the W Iberian margin by alignment of five tie points that represent main trends changes in the *G. bulloides* δ^18^O_plank_ curves (Supplementary Fig. 12) as this species and record has previously been shown to be useful stratigraphical tool in this region to synchronize their signal to Greenland climate evolution^94–96^. The resulting marine chronologies yield coherent main structures in alkenone-SST records^92,97^ and δ^18^O_sw_ records^18,59^ of each site (Supplementary Figs. 13 and 14), but ^18^O_sw_ on the W Iberian Margin (MD01-2444) is somewhat attenuated, potentially due to low biogenic productivity and foraminiferal growth periods which are biased against extreme conditions^96,98^.

### Tuning of subpolar-polar North Atlantic marine age models for Iberian Margin to speleothem chronology

For ODP 983^99^ and 984^100^ we align the major reduction in %*N. pachyderma sinistral* with the deglacial temperature rise in the Alkenone SST record from ODP 977, a clear structure that correlates well all along the Iberian margin cores, as well as a subsequent local maximum in %*N. pachyderma sinistral* evident as a local minimum in alkenone SST (Supplementary Fig. 14). The strategy is based on the synchronicity of millennial scale cooling events resulting from AMOC reorganization, widely used to apply an Antarctic or synthetic Greenland chronology to marine records^99^. Our tuning is consistent with δ^18^O_plank_ and IRD records (Supplementary Fig. 14). We align the Eirik Drift core MD03-2664^101,102^ by correlating two major structures in the %*N. pachyderma sinistral* with ODP 984 (Supplementary Fig. 14), which results in end of IRD deposition nearly synchronous with the other N Atlantic sites. The δ^18^O_sw_ at this location should have a strong regional signature of the NAIS melting through the Labrador Sea, and the major melting is observed after the main NE Atlantic freshening as recorded in the NW Iberian δ^18^O_NISA_ (Supplementary Fig. 13), but coincident with one of the minor freshening structures of the NISA record at the onset of the MIS 5e and with comparable amplitude. The benthic record δ^18^O_benthic_ at these shallow N Atlantic sites (e.g., 984 1650 m, 983 1985 m) is known to lead that of deep N Atlantic and S Atlantic and Pacific over the TI^103^ and the applied tuning suggests a similar pattern in the TII (Supplementary Fig. 15).

Within H11, we have not relied on temperature records for tuning, because the alkenone temperatures exhibit a slower rate of warming than implied by δ^13^C_NISA_. Additionally, compared to alkenones, Mg/Ca SST records from the same cores (not shown)^18,91,93^ exhibit divergent millennial structure and amplitude of SST change across H11 likely reflecting a seasonality control in the H11 temperature signal. More intense winter cooling but summer warming^104^ during cold reversals may lead to differences in rate of winter vs annual or summer warming, which may enhance discrepancies among proxies which may have differing influence of summer and winter seasons. An additional factor which has been documented to slow the manifestation of deglacial SST rise in alkenone records on the Iberian Margin is the transport of glacial-aged alkenones into sediments of deglacial and early interglacial age^29^.

### Tuning of Western Caribbean marine age models for Iberian Margin to speleothem chronology

We tune ODP 999 using its δ^18^O_sw_ record during the termination and using its δ^18^O_planktic_ record during the interglacial. δ^18^O_sw_ tuning of TII is based on observed lags during TI. During TI, the δ^18^O_sw_ of ODP 999^67^ is based on 14 C age model, updated to INTCAL2013 with constant 400 year reservoir age. During TI the W Iberian margin δ^18^O_benthic_^105^ is on chronology tuned to GRIP^106^ and Hulu^90^. We observe that depletion of δ^18^O_sw_ at ODP 999 lags that of W Iberian margin δ^18^O_benthic_ by an average of 1100 years (Supplementary Fig. 17a). This lag is consistent with timescales of ocean mixing required to distribute the ice volume signal^107^. Therefore the same lag is used on TII to align the δ^18^O_sw_ and W Iberian margin δ^18^O_benthic_^105^ and assign chronology to ODP 999. We do not use the 999 δ^18^O_planktic_ for tuning during the termination because during the phase of meltwater addition, δ^18^O_planktic_ would be expected to have contrasting temperature components on the Iberian margin (strong cooling linked to AMOC shutdown) and western Caribbean (no cooling from AMOC shutdown). However, during the interglacial, these two subtropical locations may be expected to have similar evolution of SST and δ^18^O_sw_.Therefore we use the combined signal of δ^18^O_planktic_ to serve directly for correlation during MIS 5e (Supplementary Fig. 17b).

### Estimation of terrestrial and marine components of the PGM EIS

We approximate the ‘marine’ proportion of the T1 and T2 Eurasian ice sheet using the ice sheet reconstructions of^46,61^ and an ice history that is the same as ICE6G for T2^108^. The sea level history and glacial isostatic adjustment modelling is from^8^, using the same ice sheet histories. ‘Marine’ ice is defined here simply as the volume of ice that is grounded below the reconstructed sea level.

### Reporting summary

Further information on research design is available in the Nature Research Reporting Summary linked to this article.

## Supplementary information

Supplementary information

Reporting Summary

## Data Availability

The absolute U-Series and Radiocarbon dates are provided in the Supplementary Information. The Speleothem stable isotope data and interpolated ages are submitted to the SISAL database, and additionally are available from the ETH Research data archive at doi: 10.3929/ethz-b-000545925.
