# Supplementary material for: Rapid northern hemisphere ice sheet melting during the penultimate deglaciation
Source: Nat Commun. 2022 Jul 2;13:3819. doi: 10.1038/s41467-022-31619-3 (PMC9250507; doi:10.1038/s41467-022-31619-3)
Supplement: Supplementary file 1 — Supplementary information [file 41467_2022_31619_MOESM1_ESM.pdf]

Supplementary Information for

Rapid Northern Hemisphere Ice Sheet Melting during the Penultimate Deglaciation

Heather M. Stoll et al.

*Correspondence to:heather.stoll@erdw.ethz.ch

STALAGMITES COVERING TI

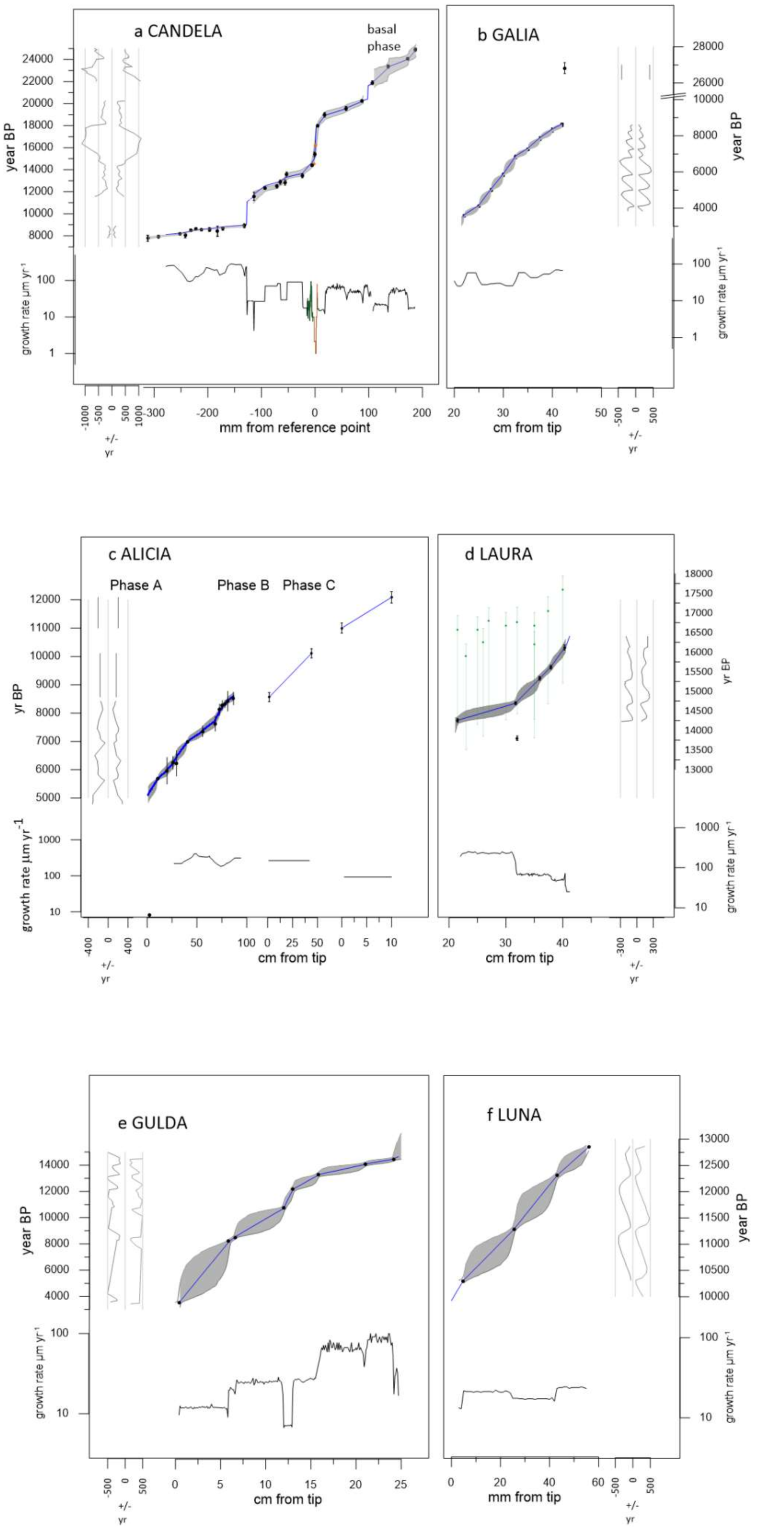

Supplementary Fig. 1 Speleothem age-depth models for stalagmites covering TI: Age-depth models (blue) of stalagmites covering Termination I. ^{230}Th dating points are shown as black dots with 2s analytical error bars and the gray band gives 95% confidence interval (CI) estimated from with the Bayesian chronology package Bchron. For each stalagmite, the width of the estimated uncertainty on age is shown adjacent to the age axis; this uncertainty is derived from Bchron 95%, or in the case of linear interpolation between two dates, it is given by the largest analytical error of the two bounding dates. a) Model for stalagmite Candela. 2 tiepoints to stalagmite Laura were used in the condensed section and are shown by orange dots. Growth rate for regions of Candela in which growth rates were estimated from width of fluorescent layers are shown with variable green line. b) Galia age model is based exclusively on ^{230}Th dates and includes a brief glacial age growth phase separated from the Holocene by a hiatus. c) Alicia has three growth phases (Phase A-C). Age-depth model for Phase A is computed with the Bayesian chronology package Bchron; in Phase B and C, age model reflects linear interpolation between basal and top date. d) Laura age model is based on five stratigraphically ordered ^{230}Th dates and excludes one ^{230}Th outlier. On stalagmite Laura, green points give calibrated ^{14}C ages assuming 0% DCF; green lines extending downward illustrate the range of calibrated ages calculated if DCF ranged from 0% (oldest possible calculated age) to 25% (youngest possible calculated age), applying formulas of [1]. e) and f) illustrate the BCHRON age models of Gulda and Luna, respectively.

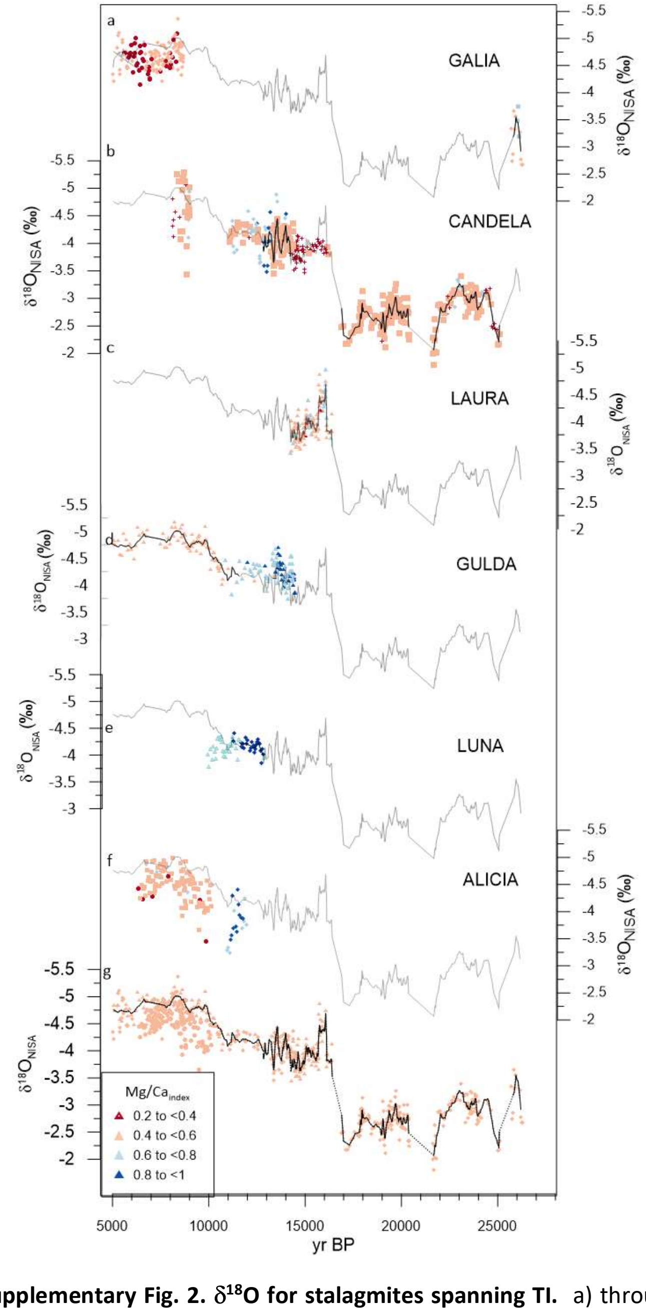

Supplementary Fig. 2. δ^{18}O for stalagmites spanning TI. a) through f) $\delta^{18}O_{NISA}$ for stalagmites Galia, Candela, Laura, Gulda, Laura, and Alicia with color coding for the Mg/Ca_{index} as shown in legend. In g), a spliced $\delta^{18}O_{NISA}$ record, and the Mg/Ca_{index} for the splice, is assembled from a succession of these stalagmites, shown as a 5 point running average. Each section of the splice is derived from a single stalagmite, and this is represented by a black line in panels a) through e), superimposed on a gray line which indicates the overall spliced record.

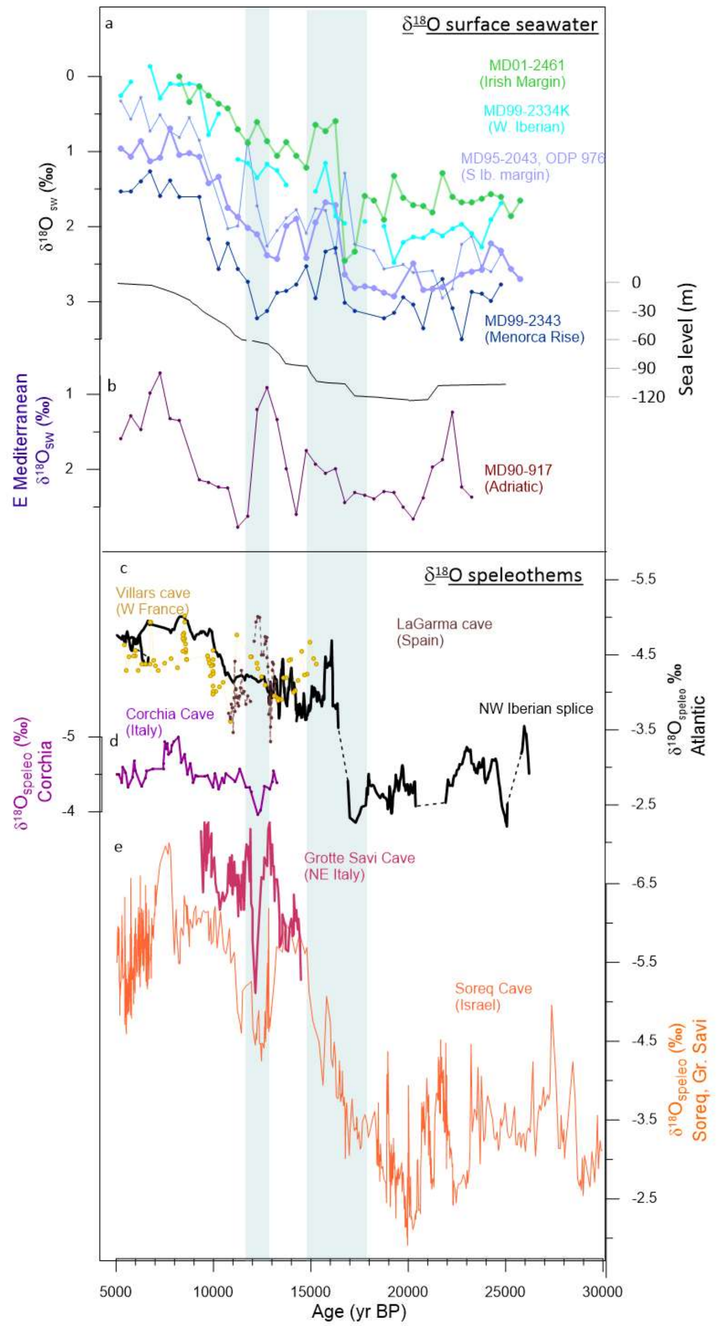

Supplementary Fig. 3. **Compilation of δ^{18}Osw and δ^{18}Ospeleothem records spanning TI**. a) $\delta^{18}O_{sw}$ from the Caribbean Sea (ODP 999)[2]and coral-based sealevel reconstruction for the TI[3]). b) $\delta^{18}O_{sw}$ from paired Mg/Ca and $\delta^{18}O_{plank}$ from *G. bulloides*, W Iberian margin[4], S Iberian margin and Menorca Rise[5], Adriatic Sea[6,7] , and Irish Margin [8]. Shown are the average 500 yr average $\delta^{18}O_{sw}$ in fixed time bins. , but results included in supplemental tables. c) Speleothem records include Villars Cave on the Atlantic Coast of France[9], our records from NW Iberia, and La Garma Cave [10] d) Corchia Cave[11] in NW Italy, e) Savi Cave in NE Italy[12], and Soreq Cave in Israel[13,14]. All records are shown on the same scale to facilitate comparison between the amplitude of $\delta^{18}O_{sw}$ and $\delta^{18}O_{speleothem,}$ This illustrates the similar amplitude and phasing of the eastern Atlantic $\delta^{18}O_{sw}$ and the $\delta^{18}O_{NISA,}$ but shows the Adriatic $\delta^{18}O_{sw}$ oscillations (~2 ‰) far smaller than the 5.5 ‰ depletion in Soreq $\delta^{18}O_{speleo}$ between the LGM and mid-Holocene, suggesting that the proximal $\delta^{18}O_{sw}$ is a minor contributor to the Soreq $\delta^{18}O_{speleo}$ changes and that other hydrological processes dominate the Soreq speleothem signal, consistent with previous comparisons of Soreq $\delta^{18}O_{speleo}$ and Eastern Mediterranean planktic $\delta^{18}O_{plank}$ [15].

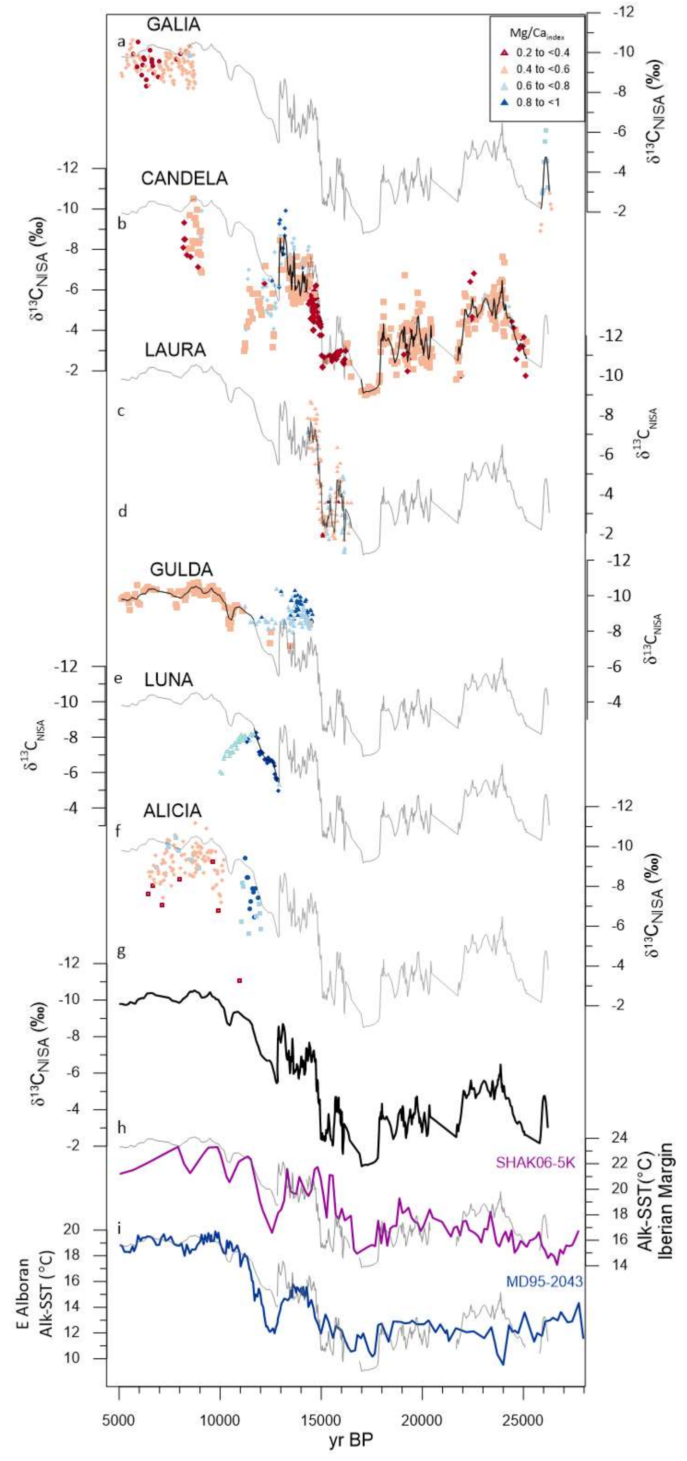

Supplementary Figure 4. $\delta^{13}C_{NISA}$ for TI speleothems. a) through f) show measured $\delta^{13}C_{NISA}$ for each stalagmite, with symbols color coded by the Mg/Ca_{index} as shown in legend. g) 5 point smoothed, spliced $\delta^{13}C_{NISA}$ (black line). Each section of the splice is derived from a single stalagmite, and this is represented by a black line in panels a) through f), superimposed on a gray line which indicates the overall spliced record. h) and i) show the SST records from west[16] and southern Iberian margin[17], in violet and blue respectively with spliced record in gray.

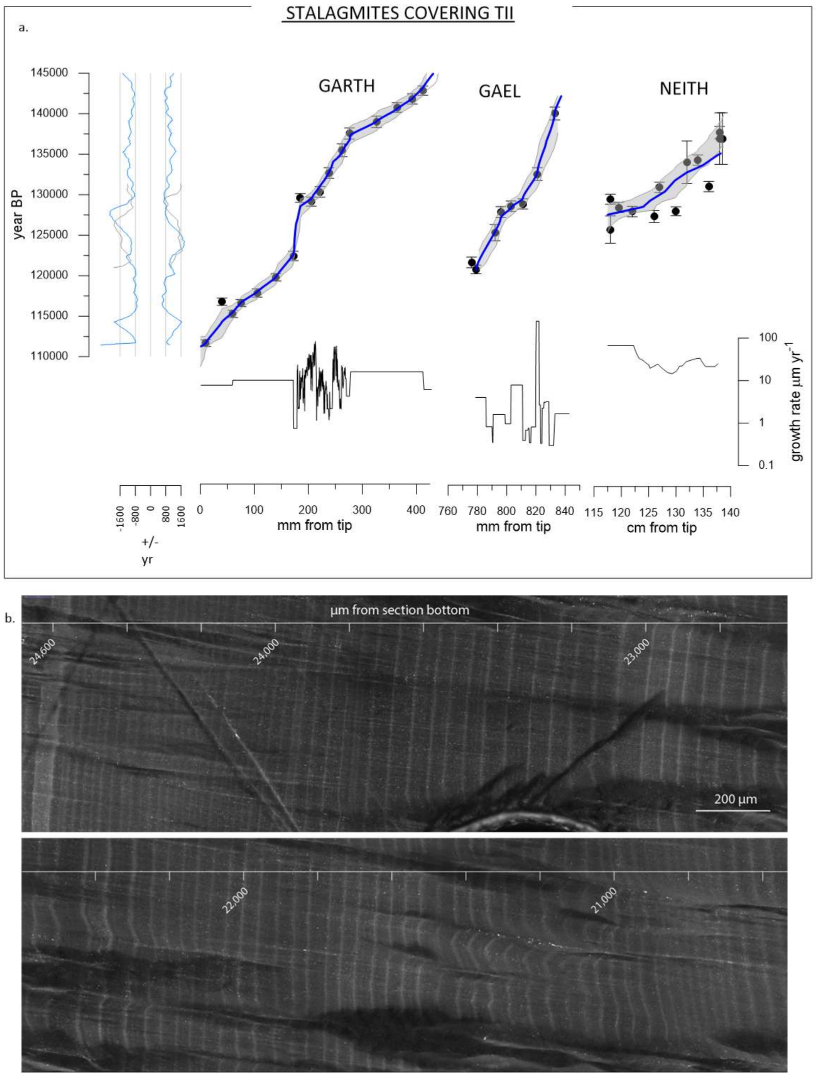

Supplementary Fig. 5 Basis for chronology of TII (following page). a) Age-depth models for stalagmites Garth, Gael, and Neith, elaborated as detailed in Supplement. Age model interpolations in Garth are based on combining ^{230}Th determination and counts of visible fluorescent laminae, age models in Gael and Neith derive from ^{230}Th. Black symbols indicate ^{230}Th age determinations, with associated analytical error bars where uncertainty exceeds size of symbol. In each age/depth plot, age model is given by blue line, and the 95% CI of BChron age model is given in gray shading. Growth rates are illustrated by thin line below each Fig.. Along the age axis, the 95% uncertainty in absolute age from Bchron model is illustrated for Garth (blue) and for Gael (gray) in the age range in which growth in Garth is condensed. b) Confocal microscope image of fluorescent growth layers in stalagmite Garth. **.** In Garth, the age model was obtained by pinning absolute chronology to the ^{230}Th date 262.5 mm from tip (135.486 ka) and progressing age upwards (and downwards)using a annual growth rates equivalent to the thickness of the annual growth layer, where it could be determined from confocal images (Fig. S9, S10). In sections where layers became too thin to count accurately, an average growth rate was selected which would match the next (stratigraphically higher) ^{230}Th age. The longest period of uncountable seasonal layering in the speleothem, reflecting the slowest speleothem growth rate, is estimated from 230^{Th} dates to span a ~2000 year period. We infer that extremely slow growth reflects cold winter conditions and propose that this duration provides a lower limit for the duration of extreme cold winter conditions linked to AMOC reduction during HE11.

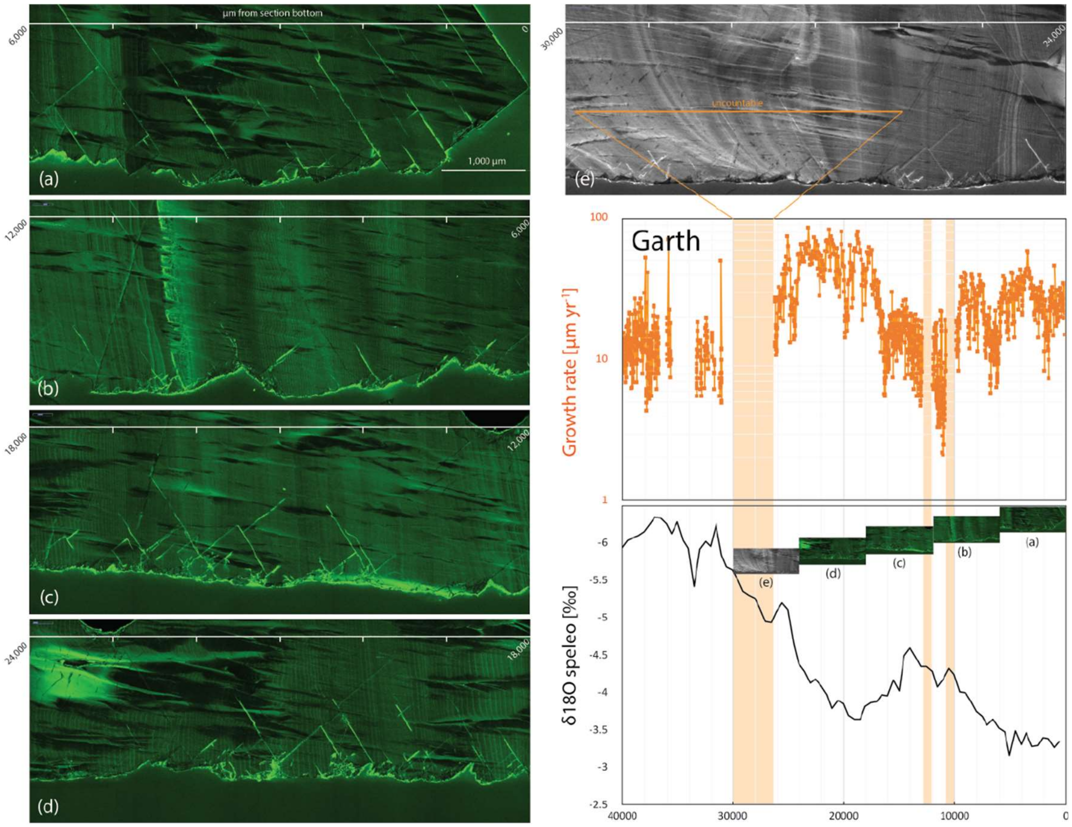

Supplementary Fig. 6. Comparison of confocal images and growth rates/oxygen isotope record for Garth. (a-e): sequential confocal images of Garth from section bottom (0 µm, a) to section top (30,000 µm, e). The lower boundary of each imaged area, where the panel letter is located, corresponds to the stalagmite material removed by micromilling a trench for isotope and trace element sampling. Note prominent parallel seasonal banding in most sections. (bottom right): growth rate (µm yr^{-1}) derived from layer counting vs section distance (note reversed axis) and $\delta^{18}O_{speleo}$, with confocal sections as insets for reference. Vertical orange windows denote areas of uncountable layers. Due to shift in growth axis along the drilling trench in the upper portion of image e) layers are condensed in the geochemical sampling in this section.

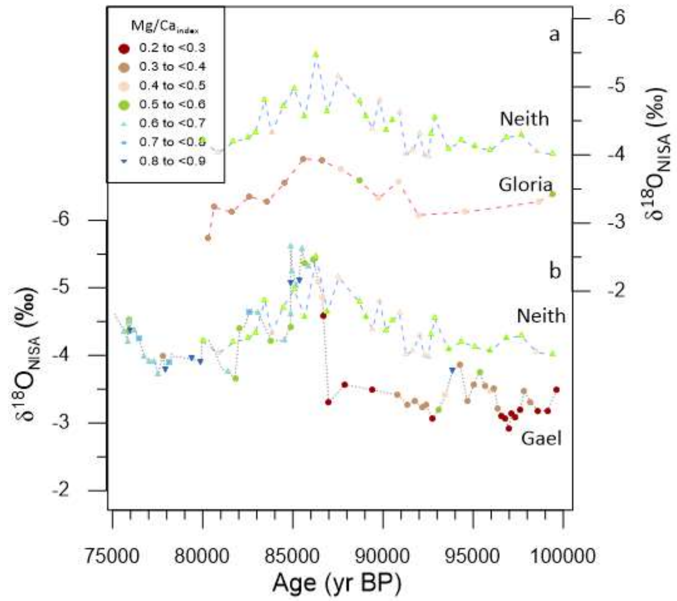

Supplementary Fig. 7. Relationship between degassing, Mg/Ca$_{index}$, and speleothem δ^{18}O records. a) $\delta^{18}O_{NISA}$ for Neith (triangles) and Gloria (crosses), color coded for the degassing Mg/Ca$_{index}$ described in Method Equation 2 as shown in legend. b) δ^{18}O for Neith (triangles) and Gael (circles), color coded for degassing Mg/Ca$_{index}$ as described in Supplementary Discussion 6. Plots use previously published age models from [18], with stalagmite Gloria plotted 1 ky older than the originally published age model to better synchronize trends.

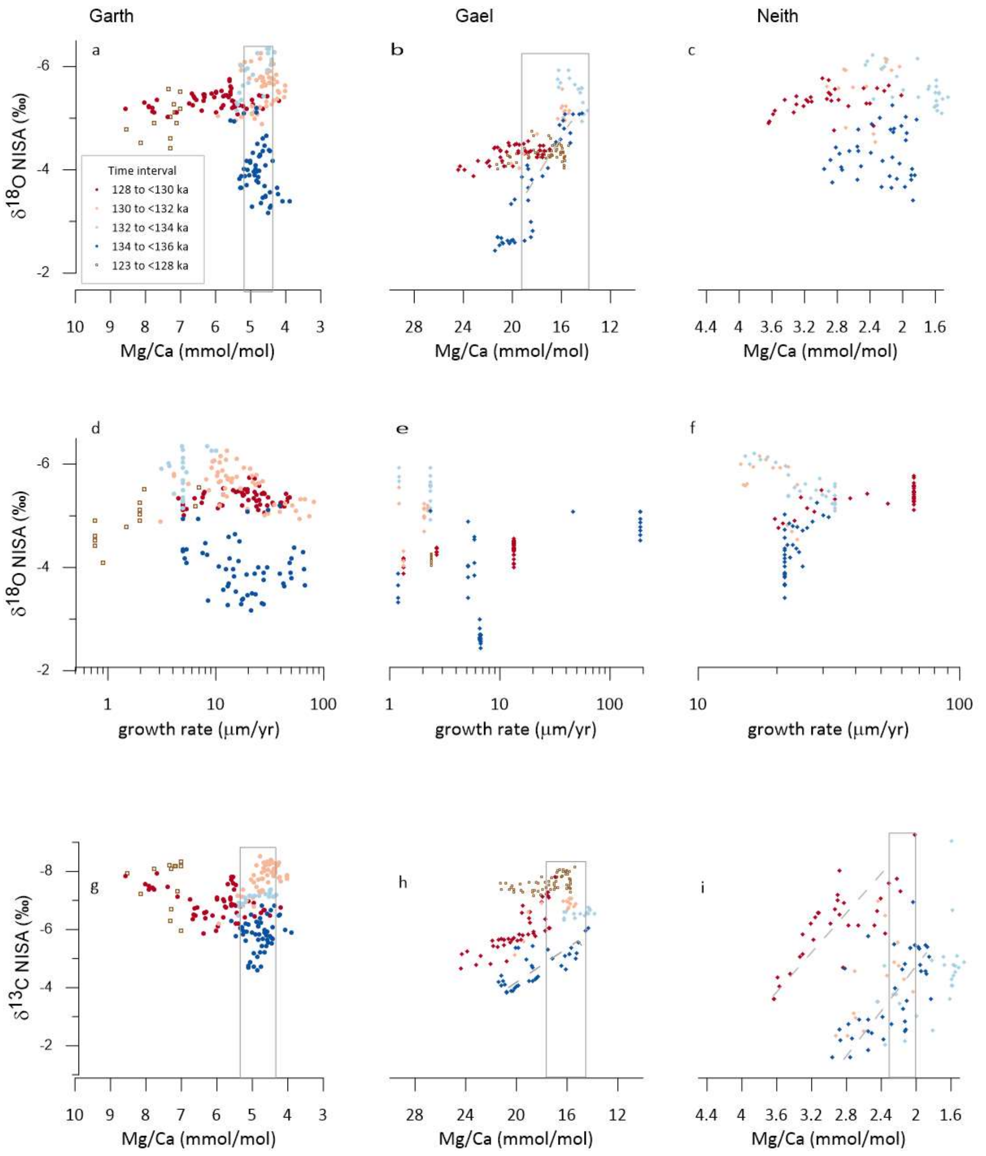

Supplementary Fig. 8. Relationship between isotope records over the TII transition and Mg/Ca and growth rate for Garth, Gael, and Neith, color coded by the time interval. a)-c) $\delta^{18}O_{NISA}$ vs Mg/Ca for each stalagmite. Gray box highlights the range of Mg/Ca during the early glacial-interglacial transition. d)-f) $\delta^{18}O_{NISA}$vs growth rate. g)-i) $\delta^{13}C_{NISA}$vs Mg/Ca for each stalagmite. Gray boxes indicate a range of $\delta^{13}C_{NISA}$for a given Mg/Ca, a range independent of PCP effects; dashed lines indicate positive correlation trajectories which may be manifestations of PCP influence on Mg/Ca and $\delta^{13}C_{speleo.}$

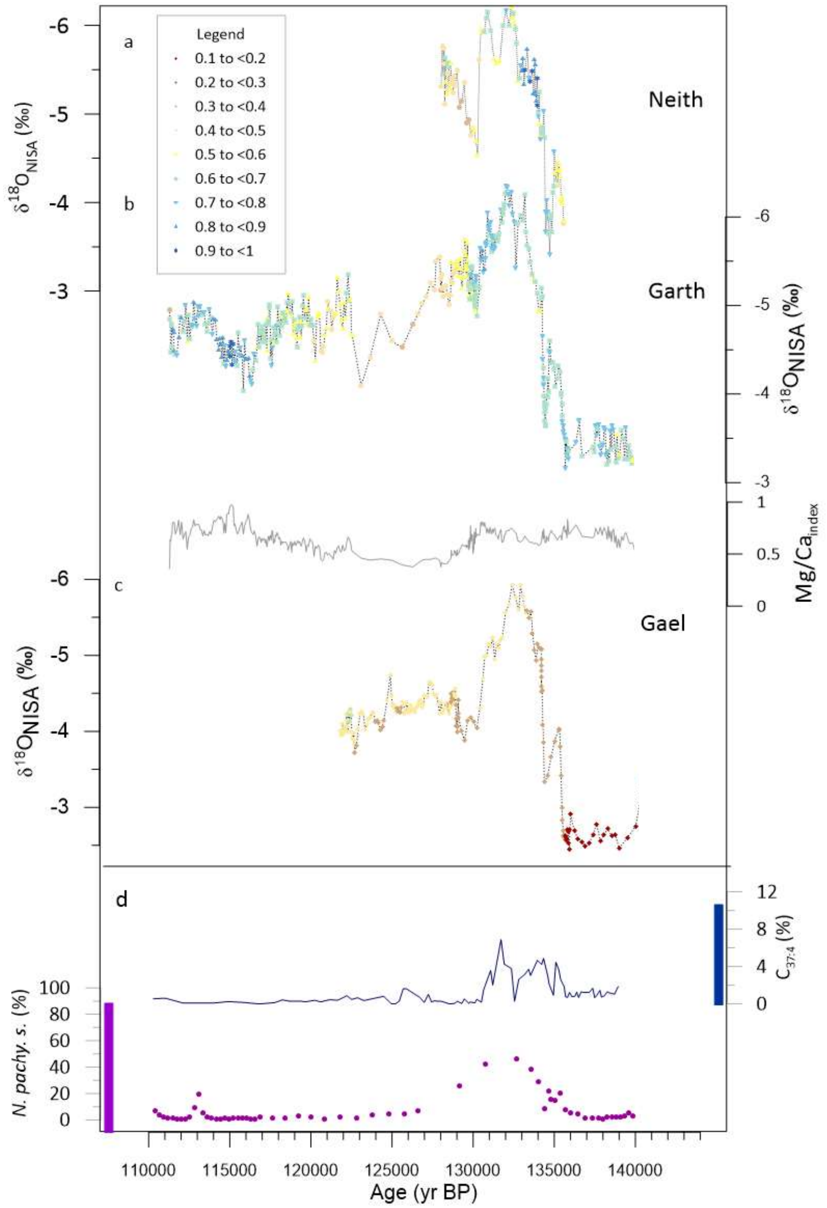

Supplementary Fig. 9. $\delta^{18}O_{NISA}$ for stalagmites spanning TII. a) through c) $\delta^{18}O_{NISA}$ for three stalagmites spanning TII (Garth, Gael, and Neith) with color coding for the degassing Mg/Ca_{index} described in Methods and as shown in legend. In b) the time series of Mg/Ca_{index} for Garth is also illustrated. d) shows two proxies for the winter climatic effect of AMOC reduction, the C37:4 % in S. Iberian margin ODP 977 [19] and the relative abundance of *N. pachyderma sinistral* in MD95-2020[20,21]. The vertical bar adjacent to each axis shows the proxy value in the same sediment archive during the HE 1 interval of TI.

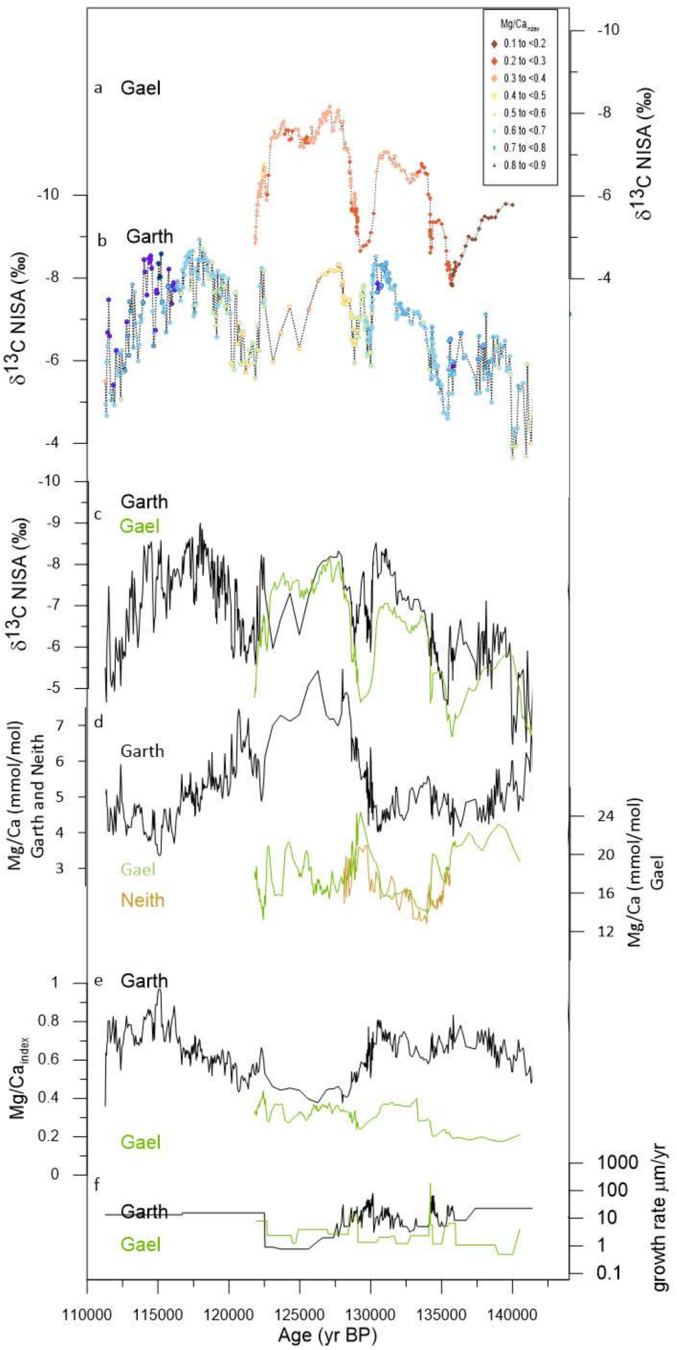

Supplementary Fig. 10. $\delta^{13}C_{NISA}$ for TII stalagmites Garth and Gael. a) and b) For Garth, and Gael, measured $\delta^{13}C_{NISA}$ with color coding for Mg/Ca_{index} as indicated in legend. c) comparison of the $\delta^{13}C_{NISA}$ records for Gael (green) and Garth (black) on the same scale. d) Mg/Ca of each stalagmite. e) Calculated Mg/Ca_{index} f)time series of growth rates.

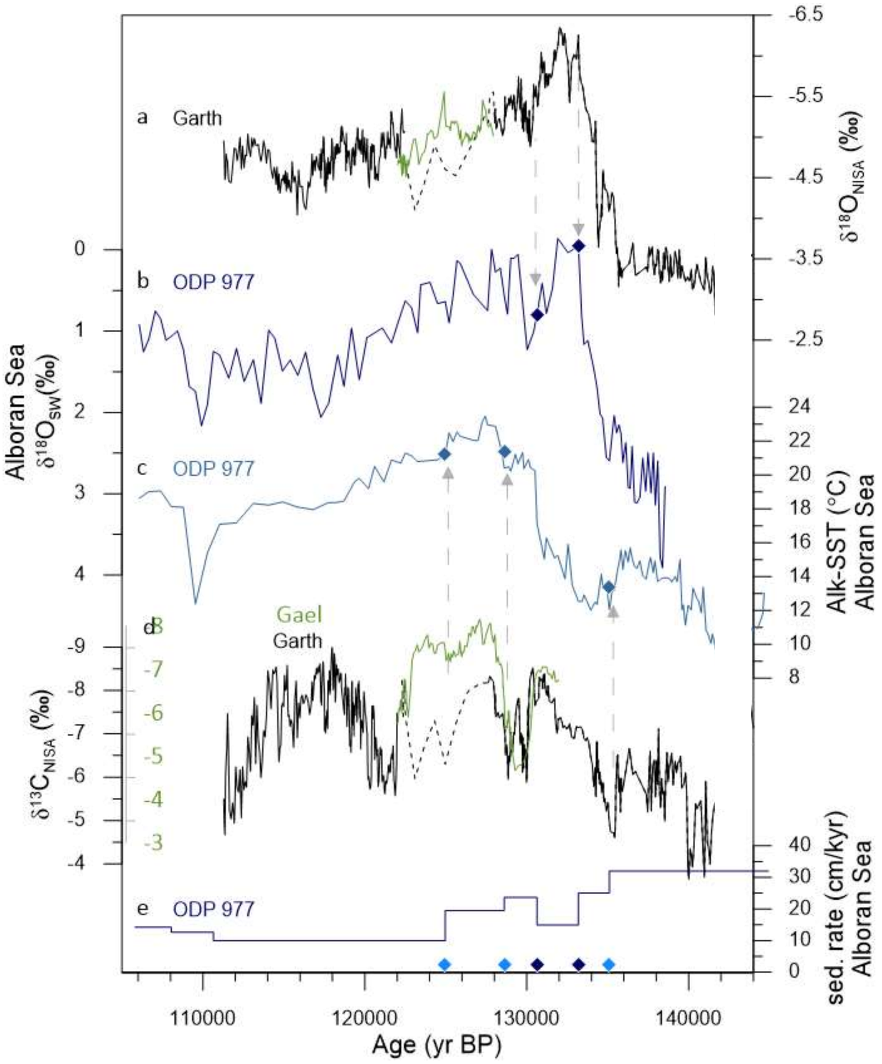

Supplementary Fig. 11. Speleothem and local marine records compared to apply absolute chronology to marine records. Alignment of the marine core ODP 977 (S. Iberian Margin) to the absolute NW Iberian speleothem chronology for the TII, as detailed in Methods. a) The new absolutely dated δ^{18}ONISAfrom NW Iberian stalagmite Garth indicating the two tie points used to synchronize the major freshening episode in the NE Atlantic with b) the $\delta^{18}O_{sw}$ record[22] of the S. Iberian Margin ODP 977, estimated through paired $\delta^{18}O_{plank}$ and Mg/Ca measurements in the planktic foraminifera *G. bulloides*.; c) Alkenone-SST reconstruction from the same ODP 977 site[23] indicating the position of the three tie points chosen to synchronize key temperature structures between the SST record and d) the $\delta^{13}C_{NISA}$record from NW Iberian stalagmite Garth, a proxy of air temperatures. e) ODP 977 sedimentation rates according to the new chronology . Diamonds below indicate the new TII tie points (blue).

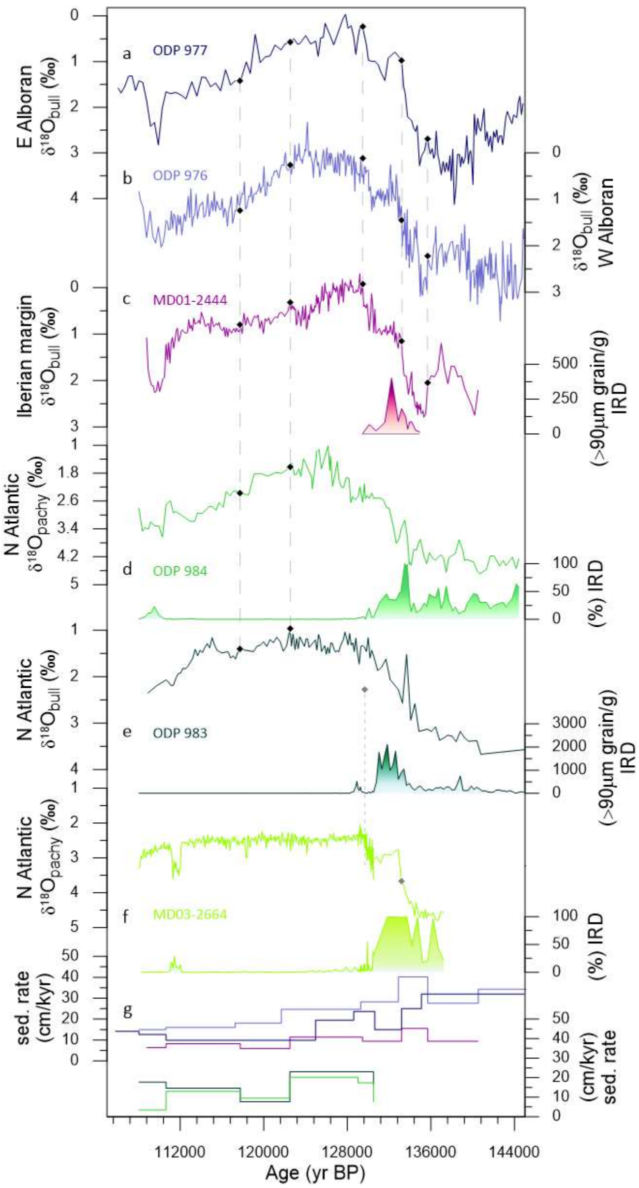

Supplementary Fig. 12. N Atlantic planktonic foraminfera $\delta^{18}O_{plank}$ records on the NW Iberian speleothem TII chronology. (previous page) a) The *G. bulloides* $\delta^{18}O_{plank}$ record[23] of the S Iberian Margin core ODP 977 in the new speleothem chronology, indicating 5 points (diamond symbols) that mark major changes in trends that can be identified along the Iberian margin cores and are used as tie points; b) The *G. bulloides* $\delta^{18}O_{plank}$ record[24] of the S Iberian margin core ODP 976; c) The *G. bulloides* $\delta^{18}O_{plank}$ record[25] of the W Iberian margin core MD01-2444 and showing the IRD record from the same core; d) The *N. pachyderma* $\delta^{18}O_{plank}$ record[26] of the N Atlantic site ODP 984, TII chronology in this core was set using the temperature records (Fig. S17) but two tie points indicated within the MIS 5e were aligned in base to the $\delta^{18}O_{plank}$ records, bellow the IRD record from the same core; e) The *G. bulloides* $\delta^{18}O_{plank}$ record[27] of the N Atlantic site ODP 983, TII chronology in this core was set in base to the temperature records (Fig. S17) but two tie points indicated within the MIS 5e were aligned in base to the $\delta^{18}O_{plank}$ records, bellow the IRD record from the same core; f) The *N. pachyderma* $\delta^{18}O_{plank}$ record[28] of the N Atlantic site MD03-2664, TII chronology in this core was set in base to the temperature records (Fig. S17) but one tie point at the middle phase of the TII freshening was used to align to the ODP 983 record, bellow the IRD[29] record from the same core; g) Sedimentation rates of the marine cores above obtained with the new tuned chronologies using the same color codes.

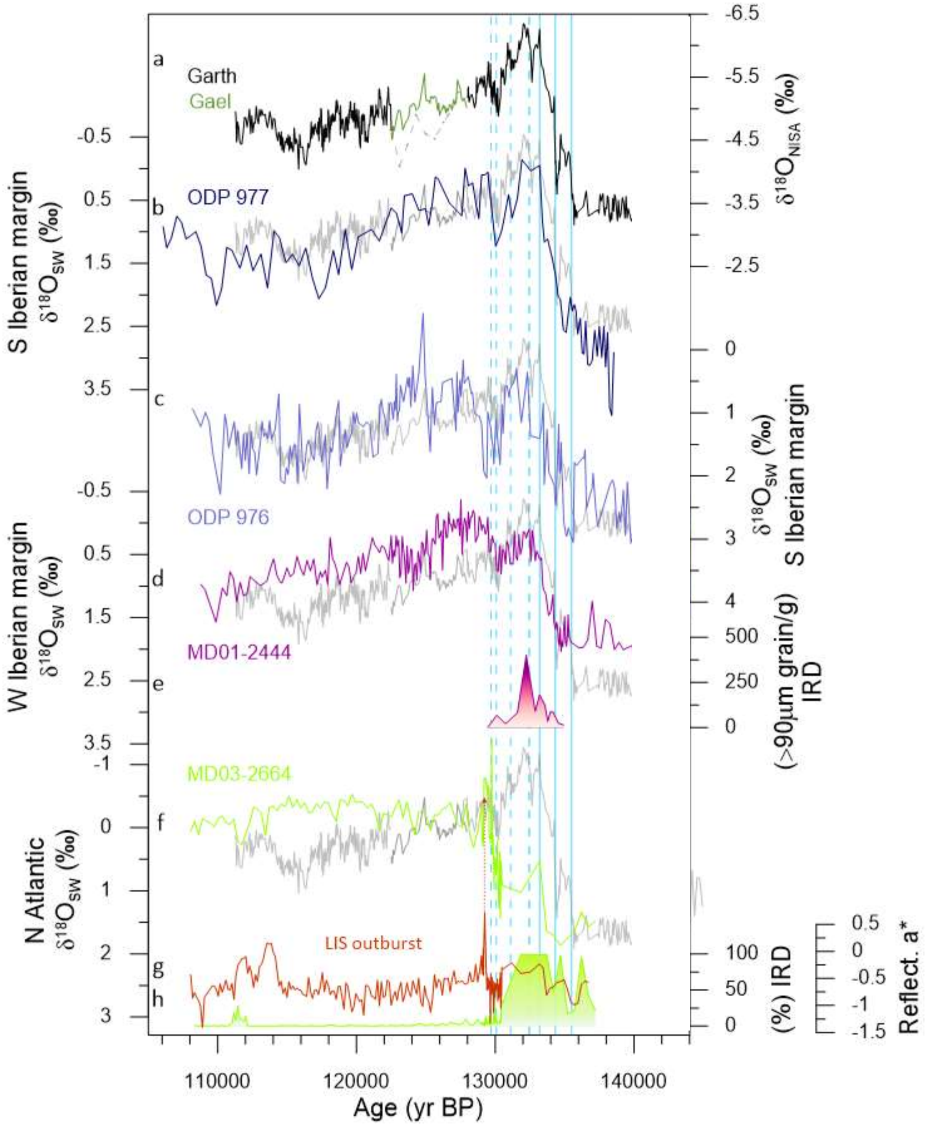

Supplementary Fig. 13 . N Atlantic records of surface $\delta^{18}O_{sw}$ freshening during TII using the tuned NW Iberian speleothem chronology. Comparison of the a) new absolutely dated $\delta^{18}O_{NISA}$record from NW Iberian stalagmites Garth and Gael, with (b,c,d) the available $\delta^{18}O_{sw}$ records around Iberia (ODP 977[22]; ODP 976[24] and MD01-2444[25]) and in the North Atlantic (f) (MD03-2664[28]) based on paired $\delta^{18}O_{plank}$ and Mg/Ca measurements in planktic foraminifera, as detailed in Methods. The record of Garth-Gael is shown in gray beneath each record. IRD records from the two N Atlantic cores MD03-2444 (e) and MD03-2664 (g) are also shown. h) The red-green optical parameter a* from core MD03-2664[30] as an indicator of a red detrital layer deposited by a NAIS outburst flood even into the Labrador Sea[31]. Blue vertical lines mark meltwater pulses.

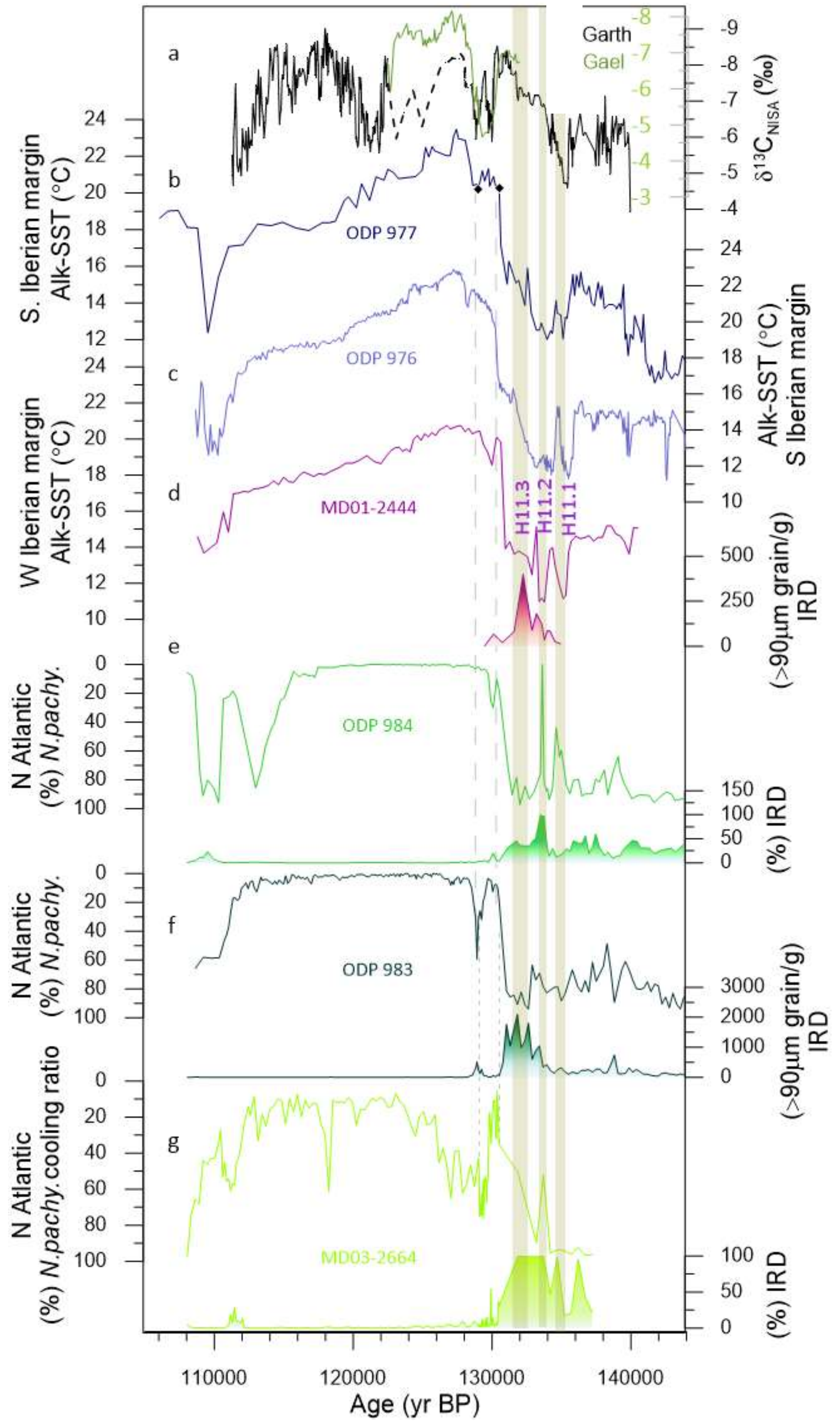

Supplementary Fig. 14. N Atlantic records of SST on the NW Iberian speleothem TII chronology. (next page) a) The new absolutely dated $\delta^{13}C_{NISA}$from NW Iberian stalagmite Garth as indicator of air temperatures in NE Atlantic. b) The Alkenone-SST record[23] of the S Iberian Margin core ODP 977 in the new Garth chronology, indicating a tie point at the end of the major TII warming phase used to tune N Atlantic core ODP 983 and 984

to ODP 977, the regional consistency on the date of this event is confirmed by the chronologies of ODP 976 and MD01-2444 cores tuned in base to their $\delta^{18}O_{plank}$. An additional tie points that marks a cooling structure in the early MIS 5e has also used to tune ODP 983 to ODP 977; c) The Alkenone-SST record[32] of the S Iberian Margin core ODP 976; d) The Alkenone-SST record[25] of the W Iberian margin core MD01-2444, showing three phases of HE11 previously distinguished on the basis of cooling events[25] e) The *N. pachyderma* % record[26] of the N Atlantic site ODP 984 indicating the tie point used to align the record to the ODP 977 one, bellow the IRD record from the same core; f) The *N. pachyderma* % record[27] of the N Atlantic site ODP 983, indicating the two tie points used to align the record to the ODP 977 one, bellow the IRD record from the same core; g) The *N. pachyderma* % record[28] of the N Atlantic site MD03-2664, indicating the two tie points used to align the record to the ODP 984 one, bellow the IRD[29] record from the same core. Pale brown vertical lines highlight the three phases of HE11 named[25] in panel d).

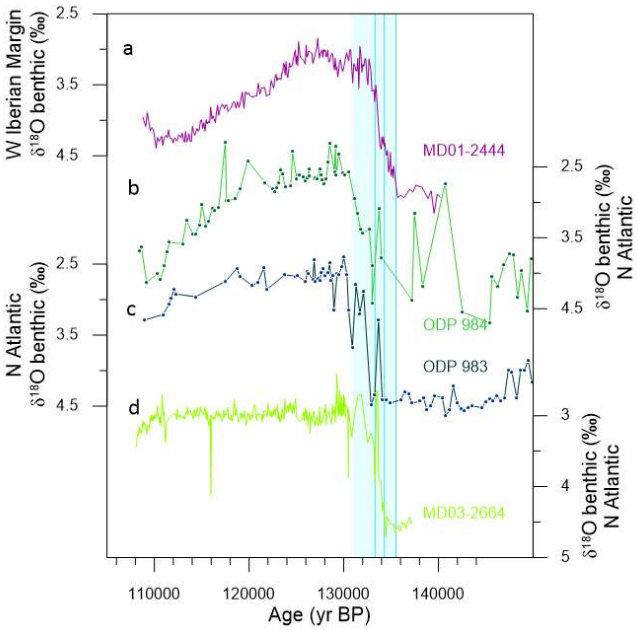

Supplementary Fig. 15. **N Atlantic records of deep water $\delta^{18}O_{benthic}$ freshening during TII using the tuned NW Iberian speleothem chronology.** a) through d) $\delta^{18}O_{benthic}$ records from the four N Atlantic cores here synchronized independently of their benthic record, MD01-2444[25], ODP 984[26], ODP 983[27] and MD03-2664[30]. Vertical blue bar indicates the period of major surface freshening in the NE Atlantic during the TII according to the Garth $\delta^{18}O_{NISA}$record. Darker blue vertical lines mark the onset of the major freshening phases.

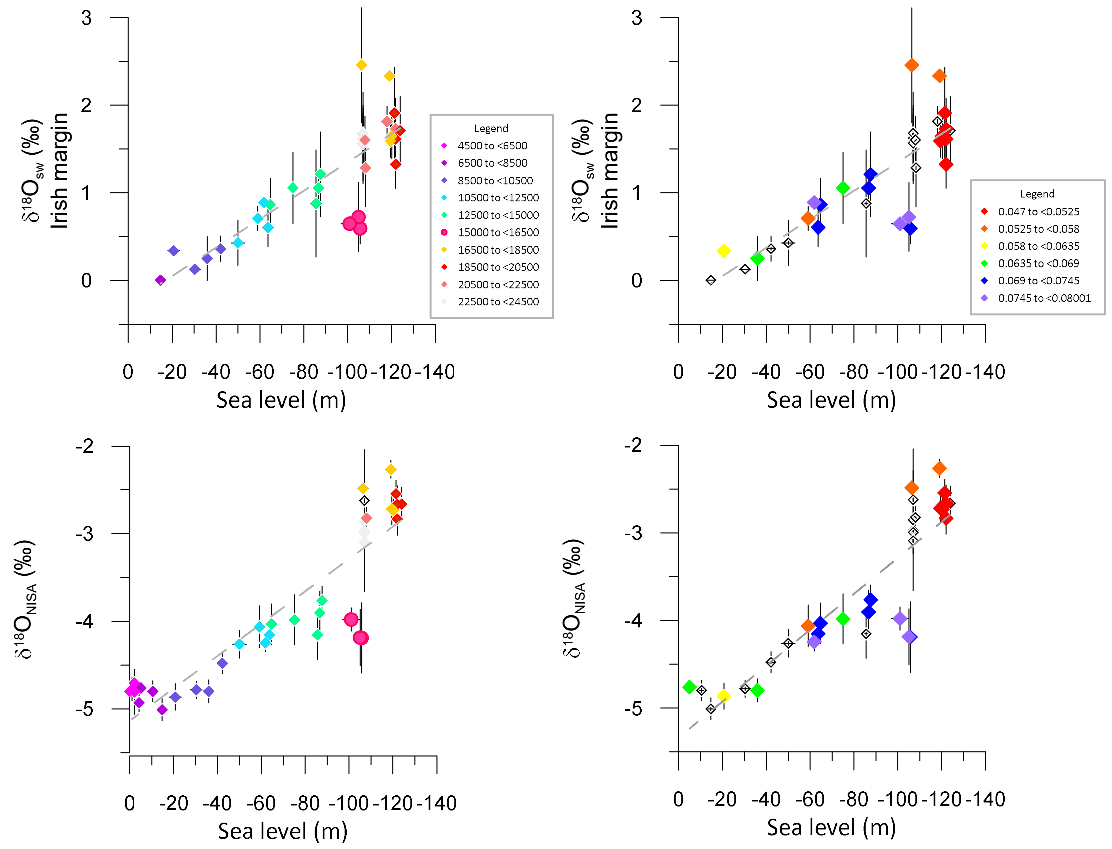

Supplementary Fig. 16 Comparison of regional freshwater anomaly over the last deglaciation. In the absence of time-varying regional surface ocean $\delta^{18}O_{sw}$ anomaly, $\delta^{18}O_{sw}$ and $\delta^{18}O_{NISA}$ would exhibit a linear relationship with sea level due to the ice volume effect. Symbols illustrate this regression on the Irish margin (upper panels), and in $\delta^{18}O_{NISA}$ (lower panels) vs global sea level (two sources; 20-12 ka [33] and 12-7 ka[3]) in fixed 500 year bins. Vertical lines illustrate plus and minus one standard deviation of the $\delta^{18}O$ data to illustrate the range of variation within each age bin. In left panels, color coding is according to sample age; in right panels, color coding is according to Pa/Th records[34].

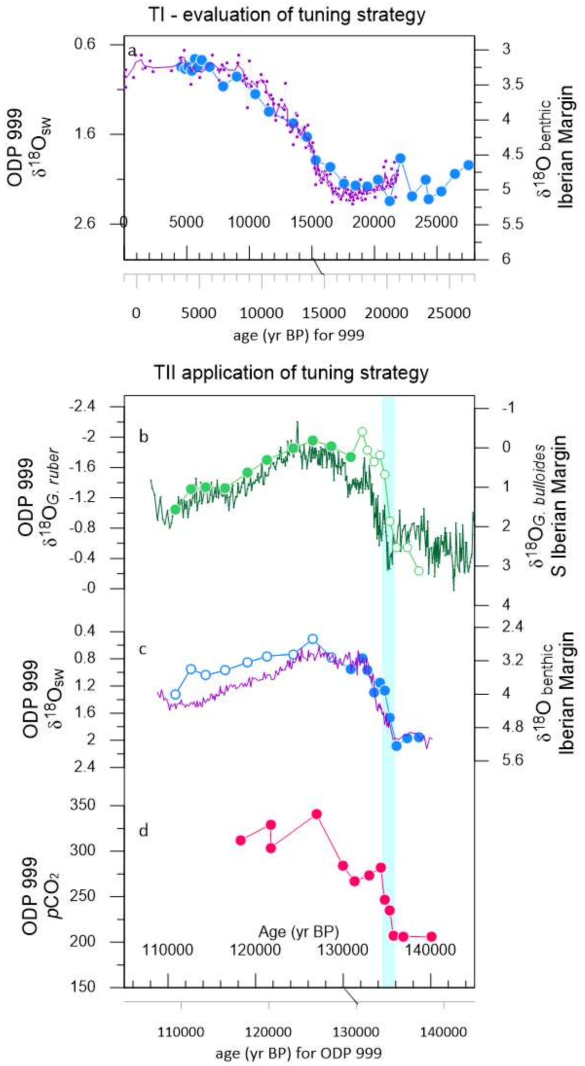

Supplementary Fig. 17. TII Tuned chronology for the Caribbean marine core ODP 999. To illustrate the proposed correlation, in a) and b)-d), two age scales are provided, an upper one for W Iberian margin core age, and the lower one for Caribbean ODP 999. . The lower age scale is in each case shifted by 1100 years to reflect the average age offset between events on W Iberian margin and at ODP 999 as observed in TI. If ocean mixing timescales during TII were similar to those of TI, then during the phase of deglacial meltwater release, the $\delta^{18}O_{sw}$ of ODP 999 should track the Iberian margin $\delta^{18}O_{benthic}$ with a similar average lag (Fig. S21c). a) Comparison $\delta^{18}O_{benthic}$ record for the W Iberia margin (purple; composite of two cores MD95-2042 & MD01-2444)[4,35,36]; and $\delta^{18}O_{sw}$ from the Caribbean Sea (ODP 999)[2] in blue. b) $\delta^{18}O_{planktic}$ for S Iberian ODP 976 (dark green line) and ODP 999 (light green circles). Filled circles indicate the time interval when this record was used for chronological alignment. c) benthic foraminifera $\delta^{18}O$ record for the W Iberia margin (purple; MD01-2444)[4,25]; and $\delta^{18}O_{sw}$ from the Caribbean Sea (ODP 999)[2] in blue circles. Filled circles indicate the time interval when this record was used for chronological alignment. d) pCO_2[37] records from the same ODP 999 site. Blue vertical line highlights interval of rapid $\delta^{18}O_{sw}$ freshening in ODP 999

Supplemental Table 1: Regression between $\delta^{18}O_{NISA}$ and $\delta^{18}O_{sw}$ over TI.

**Location (n,** age bins with data)	**r^2**	**Std error**	**slope**	**UL slope**	**LL slope**	**p-value**
**Irish Margin (31)**	**0.91**	**0.19**	0.73	0.82	0.65	5.9E-17
**S Iberian Margin MD 2443(39)**	**0.85**	**0.28**	0.74	0.84	0.63	9.2E-17
**W. Iberia Margin 2334K(36)**	**0.85**	**0.33**	0.84	0.96	0.71	1.2E-15
Florida Straits (33)	0.74	0.36	0.69	0.84	0.55	1.7E-10
Menorca Rise (38)	0.68	0.40	0.64	0.78	0.49	2.6E-10
S. Iberian ODP 976 (37)	0.67	0.50	0.78	0.97	0.59	6.9E-10
Adriatic (37)	0.32	0.47	0.35	0.53	0.17	2.9E-04

Supplemental Table 2: Regression between Irish Margin $\delta^{18}O_{sw}$ and the $\delta^{18}O_{sw}$ in other regions

Location	**r^2 (* for p value <0.05)**
S. Iberian (2443)	0.77*
W. Iberian (2334K)	0.77*
Menorca Rise	0.52*
S. Iberian ODP 976	0.33*
Florida Straits	0.60*
Adriatic	0.09

Supplemental Table 3: Regression analysis of speleothem $\delta^{18}O$ with $\delta^{18}O_{sw}$ (reporting r^2 ; bold indicates where p-value <0.05)

	Corchia (13.5-3 ka BP)	Grotte Savi (15.5-9.5 ka)	NISA 13.5-3 kaBP
**Irish Margin**	**0.27**	0.14	**0.81**
**S Iberian Margin**	**0.20**	0.10	**0.86**
**W. Iberia Margin**	**0.36**	0.37	**0.83**
Florida Straits	0.19	0.24	0.46
Menorca Rise	0.26	0.21	0.81
ODP 976	0.04	0.05	0.76
Adriatic	0.01	0.00	0.08

Uranium and thorium isotopic compositions and ^{230}Th ages for samples analyzed by ICP-MS.

Sample	Distance	^{238}U	^{232}Th	d^{234}U	[^{230}Th/^{238}U]	[^{230}Th/^{232}Th]	^{230}Th Age (yr)	^{230}Th Age (yr BP)	d^{234}U$_{initial}$
ID	from reference depth (mm)	(ppb)	(ppt)	(measured[a])	(Activity[b])	(ppm[c])	(uncorrected)	(corrected[b,d,e])	(corrected)
**CANDELA-TOP**	-312	131.8 ±0.3	426.3 ±9.3	33.9 ±3.7	0.073 ±0.002	370 ±15	7933 ±273	**7782 ±277**	35 ±4
**CANDELA-20**	-292	231.8 ±0.4	869.6 ±17.5	30.1 ±1.4	0.0737 ±0.0009	324 ±8	8098 ±103	**7926 ±127**	31 ±1
**CANDELA-60**	-252	207.4 ±0.4	185.1 ±3.7	31.8 ±1.6	0.0751 ±0.0006	1388 ±30	8242 ±67	**8150 ±70**	33 ±2
**CANDELA-70**	-242	140.4 ±0.3	154.9 ±6.6	33.3 ±3.4	0.074 ±0.002	1109 ±55	8130 ±215	**8039 ±215**	34 ±3
**CANDELA-80**	-232	161.1 ±0.2	311.5 ±6.3	32.4 ±1.4	0.0786 ±0.0009	670 ±15	8633 ±99	**8512 ±106**	33 ±1
**CANDELA-90**	-222	135.3 ±0.3	208.5 ±3.9	32.7 ±2.5	0.080 ±0.001	850 ±19	8729 ±120	**8626 ±122**	34 ±3
**CANDELA-100**	-212	198.4 ±0.3	226.3 ±4.6	34.3 ±1.5	0.0790 ±0.0006	1142 ±24	8656 ±68	**8558 ±72**	35 ±2
**CANDELA-115**	-197	178.3 ±0.3	906.3 ±18.2	31.6 ±1.4	0.080 ±0.001	259 ±6	8784 ±127	**8574 ±162**	32 ±1
**CANDELA-130**	-182	191.0 ±0.3	3611.8 ±72.5	28.3 ±1.6	0.082 ±0.002	71 ±2	9019 ±260	**8417 ±459**	29 ±2
**CANDELA-140**	-172	149.6 ±0.4	54.4 ±3.2	26.6 ±2.5	0.079 ±0.001	3584 ±217	8730 ±139	**8659 ±139**	27 ±3
**CANDELA-180**	-132	112.1 ±0.2	116.7 ±2.6	35.9 ±2.5	0.082 ±0.001	1301 ±37	9008 ±172	**8919 ±173**	37 ±3
**CANDELA-185**	-114	91.0 ±0.3	133.4 ±7.5	159.3 ±5.8	0.118 ±0.003	1327 ±83	11677 ±356	**11580 ±356**	165 ±6
**CANDELA-205**	-94	238.5 ±0.5	329.5 ±4.1	88.7 ±2.3	0.118 ±0.001	1401 ±21	12427 ±114	**12330 ±115**	92 ±2
**CANDELA-220**	-71	165.3 ±0.3	66.5 ±4.3	78.5 ±2.1	0.118 ±0.001	4819 ±315	12567 ±160	**12496 ±160**	81 ±2
**CANDELA-225**	-65	161.1 ±0.3	147.9 ±3.4	81.1 ±2.5	0.121 ±0.002	2179 ±58	12957 ±186	**12873 ±186**	84 ±3
**CANDELA-232**	-56	109.2 ±0.5	51.7 ±6.9	68.2 ±5.9	0.119 ±0.002	4158 ±562	12907 ±233	**12834 ±233**	71 ±6
**CANDELA-235**	-53	126.3 ±0.4	575.9 ±7.5	72.3 ±4.6	0.127 ±0.002	460 ±9	13751 ±218	**13567 ±226**	75 ±5
**CANDELA-245**	-24	151.6 ±0.5	224.1 ±7.6	112.4 ±5.3	0.130 ±0.002	1453 ±52	13560 ±199	**13461 ±200**	117 ±5
**CAN-EDX**	-6	176.1 ±0.3	444.1 ±9.0	150.8 ±1.7	0.144 ±0.001	942 ±20	14541 ±83	**14409 ±94**	157 ±2
**CANEDm3**	-3	261.8 ±0.3						**15662 ±103**	
**CANDELA-270**	-1	200.8 ±0.5	1238.5 ±6.0	165.7 ±2.2	0.156 ±0.002	417 ±5	15603 ±178	**15390 ±194**	173 ±2
**CAN-ED4.5**	5	220.9 ±0.3	213.8 ±4.5	229.8 ±1.7	0.189 ±0.001	3218 ±69	18092 ±76	**18000 ±78**	242 ±2
**CANDELA-290**	18	156.7 ±0.4	1495.6 ±6.0	219.6 ±2.8	0.199 ±0.002	343 ±3	19271 ±170	**18985 ±204**	232 ±3
**CANDELA-330**	58	187.5 ±0.4	499.8 ±3.7	162.2 ±2.2	0.193 ±0.002	1192 ±13	19685 ±186	**19559 ±189**	171 ±2
**CANDELA-360**	88	237.0 ±0.5	359.8 ±7.3	170.4 ±1.9	0.200 ±0.001	2170 ±45	20324 ±105	**20218 ±108**	180 ±2
**CANDELA-B10**	107	239.1 ±0.3	2036.9 ±40.9	230.6 ±2.0	0.228 ±0.001	441 ±9	22168 ±88	**21900 ±166**	245 ±2
**CANDELA-B38**	136	274.2 ±0.6	163 ±4	152.8 ±2.8	0.2240 ±0.0011	6212 ±139	23442 ±146	**23356 ±147**	163 ±3
**CANDELA-B90**	173	256.3 ±0.4	750.2 ±15.1	137.3 ±1.6	0.227 ±0.001	1280 ±26	24195 ±99	**24051 ±112**	147 ±2
**CANDELA-B92**	187	267.6 ±0.4	571 ±12	170.2 ±2.2	0.2412 ±0.0012	1865 ±39	25027 ±154	**24904 ±159**	183 ±2
**ALICIA-10**	10	43.2 ±0.1	2252.7 ±10.9	1397.4 ±9.7	0.120 ±0.003	38 ±1	5590 ±155	**4900 ±351**	1417 ±10
**ALICIA-105**	105	73.2 ±0.1	444.2 ±8.9	1484.0 ±2.5	0.1294 ±0.0007	352 ±7	5807 ±33	**5677 ±60**	1508 ±3
**ALICIA-200**	200	63.4 ±0.1	3958.9 ±79.3	1694.5 ±2.9	0.1614 ±0.0008	43 ±1	6693 ±36	**5964 ±474**	1724 ±4
**ALICIA-260**	260	57.3 ±0.2	2001.6 ±10.3	1597.0 ±8.4	0.156 ±0.003	73 ±1	6695 ±123	**6247 ±229**	1626 ±9
**ALICIA-295**	295	52.5 ±0.1	2431.2 ±48.7	1582.7 ±2.9	0.157 ±0.006	56 ±2	6811 ±264	**6228 ±451**	1611 ±4
**ALICIA-403**	403	52.2 ±0.1	260.3 ±5.3	1399.4 ±3.0	0.1524 ±0.0009	504 ±11	7111 ±44	**6991 ±61**	1428 ±3
**ALICIA-557**	557	39.5 ±0.0	862.6 ±17.3	1409.9 ±2.8	0.165 ±0.001	124 ±3	7681 ±58	**7360 ±193**	1440 ±3
**ALICIA-685**	685	46.1 ±0.1	1094.7 ±21.9	1397.3 ±3.0	0.170 ±0.004	118 ±3	7979 ±173	**7628 ±266**	1428 ±3
**ALICIA-727**	727	38.1 ±0.0	622.5 ±12.5	1412.8 ±3.2	0.180 ±0.003	181 ±5	8380 ±151	**8120 ±204**	1446 ±3
**ALICIA-755**	755	50.8 ±0.1	680.3 ±13.6	1399.9 ±2.3	0.181 ±0.003	223 ±5	8504 ±126	**8278 ±169**	1433 ±2
**ALICIA-782**	782	49.9 ±0.1	197.4 ±2.2	1434.8 ±6.7	0.183 ±0.003	761 ±14	8432 ±128	**8326 ±131**	1469 ±7
**ALICIA-810**	810	46.0 ±0.0	1275.4 ±25.6	1429.8 ±2.2	0.190 ±0.005	113 ±4	8817 ±264	**8423 ±351**	1464 ±3
**ALICIA-867**	867	45.6 ±0.0	834.7 ±17.0	1448.3 ±2.3	0.191 ±0.003	172 ±5	8795 ±164	**8515 ±223**	1484 ±3
**ALICIAB-15**	15	42.9 ±0.2	462.1 ±3.2	1497.9 ±8.3	0.194 ±0.003	297 ±5	8749 ±148	**8565 ±161**	1535 ±9
**ALICIAB-440**	440	52.1 ±0.1	227.4 ±2.3	1600.0 ±7.0	0.235 ±0.003	887 ±16	10219 ±159	**10111 ±160**	1647 ±7
**ALICIAC-TIP**	0	33.60 ±0.04	668.1 ±13.4	1603.8 ±4.1	0.259 ±0.002	214 ±5	11288 ±92	**11002 ±179**	1655 ±4
**ALICIAC-90**	90	40.8 ±0.1	11797.9 ±58.8	1834.2 ±7.4	0.307 ±0.004	248 ±4	12352 ±174	**12088 ±202**	1898 ±8
**GALIA-20**	20	163.2 ±0.4	245.8 ±2.1	95.6 ±3.4	0.0098 ±0.0003	108 ±3	983 ±25	**883 ±32**	96 ±3
**GALIA-30**	30	163.6 ±0.4	63.4 ±2.4	96.7 ±3.3	0.0104 ±0.0003	444 ±21	1041 ±31	**971 ±31**	97 ±3
**GALIA-40**	40	162.5 ±0.4	34.1 ±2.2	93.8 ±4.0	0.0111 ±0.0003	876 ±60	1117 ±28	**1051 ±28**	94 ±4
**GALIA-50**	50	163.5 ±0.6	21.5 ±1.9	95.5 ±6.2	0.0123 ±0.0002	1546 ±138	1233 ±26	**1170 ±26**	96 ±6
**GALIA-60**	60	201.7 ±0.8	176.6 ±2.3	97.5 ±4.8	0.0124 ±0.0003	233 ±7	1239 ±32	**1155 ±34**	98 ±5
**GALIA-70**	70	172.6 ±0.7	24.0 ±2.3	84.1 ±7.1	0.0124 ±0.0003	1465 ±142	1252 ±32	**1188 ±32**	84 ±7
**GALIA-80**	80	163.1 ±0.6	98.0 ±1.9	96.2 ±5.0	0.0157 ±0.0003	430 ±12	1570 ±34	**1494 ±35**	97 ±5
**GALIA-100**	100	146.4 ±0.3	46.4 ±1.9	95.9 ±2.9	0.0169 ±0.0004	877 ±41	1689 ±38	**1621 ±38**	96 ±3
**GALIA-110**	110	120.8 ±0.4	27.4 ±1.9	104.4 ±6.3	0.0177 ±0.0004	1287 ±93	1765 ±39	**1699 ±39**	105 ±6
**GALIA-120**	120	135.7 ±0.5	201.0 ±2.3	95.6 ±5.0	0.0181 ±0.0004	201 ±5	1814 ±45	**1714 ±49**	96 ±5
**GALIA-150**	150	116.4 ±0.5	31.2 ±1.9	96.3 ±5.4	0.0197 ±0.0005	1213 ±80	1978 ±50	**1911 ±50**	97 ±5
**GALIA-175**	175	153.1 ±0.6	119.5 ±2.6	96.6 ±6.8	0.0239 ±0.0005	505 ±15	2404 ±52	**2323 ±53**	97 ±7
**GALIA-200**	200	199.3 ±1.1	2484.6 ±16.7	97.6 ±7.7	0.032 ±0.002	42 ±2	3174 ±177	**2783 ±242**	98 ±8
**GALIA-220**	220	207.8 ±0.9	308.7 ±3.2	105.0 ±7.5	0.0366 ±0.0006	407 ±7	3674 ±65	**3575 ±67**	106 ±8
**GALIA-250**	250	211.9 ±0.9	277.6 ±2.4	98.0 ±5.8	0.0414 ±0.0004	522 ±7	4193 ±51	**4098 ±54**	99 ±6
**GALIA-275**	275	186.2 ±1.0	283.9 ±2.5	109.1 ±9.1	0.0508 ±0.0006	549 ±8	5108 ±79	**5008 ±81**	111 ±9
**GALIA-300**	300	238.1 ±1.0	996.8 ±3.5	98.5 ±5.6	0.0590 ±0.0006	232 ±2	6014 ±74	**5843 ±92**	100 ±6
**GALIA-325**	325	215.2 ±1.3	50.5 ±3.0	102.7 ±9.5	0.0676 ±0.0008	4752 ±288	6891 ±103	**6825 ±103**	105 ±10
**GALIA-350**	350	264.8 ±0.8	429.7 ±2.5	101.4 ±3.9	0.0719 ±0.0005	731 ±7	7359 ±63	**7256 ±67**	104 ±4
**GALIA-375**	375	197.5 ±1.1	155.8 ±2.4	112.1 ±8.5	0.0781 ±0.0008	1632 ±28	7927 ±103	**7847 ±103**	115 ±9
**GALIA-400**	400	210.4 ±1.2	128.5 ±2.8	118.2 ±9.3	0.0829 ±0.0009	2240 ±53	8393 ±116	**8317 ±116**	121 ±10
**GALIA-420**	420	209.2 ±1.0	172.8 ±2.3	112.7 ±5.8	0.0853 ±0.0008	1704 ±27	8689 ±96	**8608 ±97**	116 ±6
**GALIA-425**	425	143.7 ±0.6	103.2 ±2.8	123.1 ±7.5	0.247 ±0.002	5662 ±157	26908 ±303	**26829 ±303**	133 ±8
**LAURA-215**	215	418.8 ±0.4	203.0 ±4.0	167.5 ±1.5	0.1442 ±0.0004	4918 ±100	14336 ±43	**14256 ±44**	174 ±2
**LAURA-32**	317	464.6 ±0.3	165.0 ±4	167.3 ±1.2	0.1483 ±0.0003	6884 ±152	14772 ±35	**14694 ±36**	174 ±1
LAURA-325	*320*	*539.3 ±1.2*	*241.0 ±4.9*	*235.1 ±2.3*	*0.148 ±0.001*	*5468 ±113*	*13880 ±58*	***13800 ±58***	*245 ±2*
**LAURA 35.5**	379	280.9 ±0.2	98.2 ±3	155.9 ±1.2	0.1528 ±0.0004	7204 ±188	15408 ±48	**15330 ±49**	163 ±1
**LAURA-390**	390	601.8 ±0.9	245.0 ±5.0	150.6 ±1.5	0.155 ±0.000	6272 ±128	15690 ±47	**15612 ±48**	157 ±2
**LAURA-420**	403	545.6 ±0.9	468.7 ±9.5	151.0 ±1.7	0.159 ±0.001	3060 ±63	16206 ±64	**16115 ±66**	158 ±2
**LUNA 3**	4.7	1998.7 ±3.0	19 ±3	-32.3 ±1.2	0.0877 ±0.0002	148517 ±22043	10369 ±29	**10300 ±29**	-33 ±1
**LUNA 24**	25.5	1038.4 ±1.1	60 ±2	-23.3 ±1.3	0.0966 ±0.0003	27384 ±1027	11355 ±35	**11285 ±35**	-24 ±1
**LUNA 42**	43	1270.7 ±1.4	96 ±3	-22.5 ±1.2	0.1049 ±0.0003	22840 ±751	12381 ±37	**12309 ±37**	-23 ±1
**LUNA 56**	56.1	1523.5 ±2.0	82 ±2	-13.2 ±1.3	0.1102 ±0.0002	33606 ±957	12920 ±34	**12849 ±34**	-14 ±1
**LUNA 62**	62.4	1148.1 ±1.6	111 ±3	2.5 ±1.4	0.2291 ±0.0005	39185 ±1112	28283 ±85	**28211 ±85**	3 ±2
**LUNA 102**	102.3	1155.7 ±1.4	27 ±2	0.7 ±1.3	0.2565 ±0.0004	181995 ±12072	32294 ±84	**32225 ±84**	1 ±1
**GUL 0-4**	0.4	240.5 ±0.3	166 ±3	-94.5 ±1.6	0.0294 ±0.0004	705 ±17	3606 ±46	**3514 ±49**	-95 ±2
**GUL-T**	5.9	373.7 ±0.6	1089 ±22	-85.5 ±1.6	0.0675 ±0.0004	382 ±8	8368 ±53	**8206 ±84**	-88 ±2
**GUL 6-8**	6.6	362.0 ±0.7	545 ±11	-83.4 ±2.2	0.0696 ±0.0003	761 ±16	8618 ±47	**8500 ±58**	-85 ±2
**GUL 12-2**	12	254.4 ±0.4	204 ±4	-74.2 ±2.1	0.0874 ±0.0004	1799 ±39	10821 ±61	**10726 ±64**	-76 ±2
**GUL-B**	13	286.7 ±0.3	215 ±5	-63.4 ±1.3	0.0994 ±0.0006	2181 ±51	12251 ±84	**12159 ±86**	-66 ±1
**GUL15-8**	15.8	294.7 ±0.4	456 ±9	-59.0 ±1.6	0.1085 ±0.0005	1156 ±24	13374 ±67	**13256 ±75**	-61 ±2
**GUL 21-6**	21	332.2 ±0.5	229 ±5	-76.8 ±1.7	0.1124 ±0.0004	2686 ±57	14179 ±64	**14087 ±66**	-80 ±2
**GUL 24-7**	24.2	339.6 ±0.4	191 ±4	-76.2 ±1.4	0.1152 ±0.0004	3375 ±73	14548 ±65	**14461 ±66**	-79 ±1

Analytical errors are 2s of the mean.

Corrected ^{230}Th ages assume the initial ^{230}Th/^{232}Th atomic ratio of $4.4 \pm 2.2 \times 10^{-6}$. Those are the values for a material at secular equilibrium, with the bulk earth ^{232}Th/^{238}U value of 3.8. The errors are arbitrarily assumed to be 50%.

U decay constants: $\lambda_{238} = 1.55125 \times 10^{-10}$ and $\lambda_{234} = 2.82206 \times 10^{-6}$. Th decay constant: $\lambda_{230} = 9.1705 \times 10^{-6}$.

[a] $\delta^{234}U = ([^{234}U/^{238}U]_{activity} - 1) \times 1000$.

[b] $[^{230}Th/^{238}U]_{activity} = 1 - e^{-l230 * T} + (d^{234}U_{measured}/1000)[l230/(l230-l234)](1 - e^{-(l230-l234)T})$, where T is the age.

[c] The degree of detrital ^{230}Th contamination is indicated by the [^{230}Th/^{232}Th] atomic ratio instead of the activity ratio.

[d] Age corrections were calculated using an average crustal ^{230}Th/^{232}Th atomic ratio of $4.4 \times 10^{-6} \pm 2.2 \times 10^{-6}$.

[e] B.P. stands for "Before Present" where the "Present" is defined as the year 1950 A.D.

Supplementary Table 4.

^{230}Th dating results for stalagmites spanning TI and the Holocene.

Sample ID	Distance from reference depth (cm)	$F^{14}C$	± (%)	14C age (yr)	± (yr)	INTCAL20 calibrated age (yr BP)	± (yr)	upper age limit	lower age limit	25% DCF corrected age (yr BP)
**LAU 3.2**	3	0.2072	±1.41	12643.00	113	**15087**	±306	15392	14781	12710
**Lau 50**	5	0.1816	±1.13	13701.64	91	**16616**	±311	16926	16305	14151
**Lau 69**	6.9	0.1911	±1.13	13293.67	91	**15984**	±273	16256	15711	13621
**Lau 10**	10	0.1866	±1.28	13485.28	103	**16259**	±324	16583	15935	13855
**Lau 15**	15	0.1845	±1.13	13577.38	91	**16391**	±304	16694	16087	13952
**LAU 21.5**	21.5	0.1819	±1.43	13689.24	115	**16603**	±363	16966	16240	14135
**Lau 23**	23	0.1929	±1.24	13218.22	99	**15890**	±297	16187	15593	13507
**Lau 250**	25	0.1816	±1.18	13703.27	95	**16619**	±318	16937	16301	14155
**Laura 26**	26	0.1866	±1.39	13484.58	112	**16252**	±353	16604	15899	13854
**Lau 27**	27	0.1777	±1.17	13877.77	94	**16802**	±272	17074	16530	14451
**Lau 30**	30	0.1798	±1.26	13783.15	101	**16705**	±312	17017	16393	14270
**LAU 32.5**	32	0.1780	±1.39	13862.84	111	**16766**	±332	17098	16434	14422
**Lau 35**	35	0.1875	±1.24	13447.36	100	**16200**	±318	16517	15882	13812
**Lau 350**	35	0.1796	±1.16	13792.60	93	**16722**	±296	17017	16426	14287
**LAURA 39**	37.4	0.1742	±1.29	14036.11	104	**17060**	±310	17370	16750	14680
**LAU 42**	40	0.1656	±1.59	14444.82	128	**17695**	±412	18106	17283	15219

Supplementary Table 5.

Radiocarbon ages for samples analyzed by AMS.

Sample	Stalagmite	Distance	^{238}U	^{232}Th	$d^{234}U$	$[^{230}Th/^{238}U]$	$[^{230}Th/^{232}Th]$	^{230}Th Age (yr)	^{230}Th Age (yr BP)	$d^{234}U_{initial}$
ID		from ref (mm)	(ppb)	(ppt)	(measured[a])	(Activity[b])	(ppm[c])	(uncorrected)	(corrected[b,d,e])	(corrected[e])
**AGAEL-67**	**GAEL**	776 ±2	194.4 ±0.3	1048.1 ±21.1	-107.7 ±1.2	0.590 ±0.001	1806 ±36	122394 ±641	**122140 ±653**	-152 ±2
**GAEL-779**	**GAEL**	779 ±2	203.5 ±0.2	174.2 ±3.5	-82.5 ±1.5	0.626 ±0.001	12048 ±244	121533 ±517	**121260 ±538**	-119 ±2
AGAEL-59	***GAEL***	*785 ±2*	*255.8 ±0.6*	*19348.3 ±389.3*	*-101.0 ±1.9*	*0.592 ±0.002*	*129 ±3*	*121089 ±1052*	***118425 ±2098***	*-141 ±3*
**AGAEL-52**	**GAEL**	792 ±2	235.0 ±0.3	8347.6 ±167.2	-86.1 ±1.1	0.619 ±0.001	287 ±6	127059 ±545	**125805 ±995**	-123 ±2
**GAEL-796**	**GAEL**	796 ±2	255.1 ±0.2	1570.2 ±31.4	-112.2 ±1.2	0.585 ±0.001	1566 ±31	128432 ±674	**128343 ±674**	-158 ±2
**AGAE-40**	**GAEL**	803 ±2	293.0 ±0.3	111.2 ±2.2	-74.2 ±1.3	0.634 ±0.001	27547 ±559	129150 ±643	**129073 ±643**	-107 ±2
**AGAE-30**	**GAEL**	811 ±2	220.7 ±0.2	174.9 ±3.5	-57.6 ±1.2	0.648 ±0.001	13482 ±272	129452 ±629	**129363 ±629**	-83 ±2
**AGAE-20**	**GAEL**	821 ±2	239.6 ±0.3	107.4 ±2.2	-52.8 ±1.5	0.661 ±0.002	24319 ±496	133114 ±786	**133035 ±786**	-77 ±2
**AGAE-10**	**GAEL**	833 ±2	326.0 ±0.4	204.5 ±4.1	-75.2 ±1.3	0.660 ±0.001	17360 ±351	140604 ±797	**140520 ±797**	-112 ±2
**GAR-10**	**GARTH**	10 ±2	276.3 ±0.3	377.6 ±7.6	134.5 ±1.4	0.740 ±0.001	8928 ±179	111764 ±404	**111671 ±405**	184 ±2
**GAR-40**	**GARTH**	40 ±2	325.3 ±0.4	525.5 ±10.6	127.4 ±1.4	0.754 ±0.001	7696 ±155	116894 ±452	**116786 ±453**	177 ±2
**GAR-60**	**GARTH**	60 ±2	277.5 ±0.3	325.2 ±6.5	146.1 ±1.4	0.762 ±0.001	10729 ±216	115383 ±444	**115286 ±445**	202 ±2
**GAR-75**	**GARTH**	75 ±2	289.5 ±0.3	229.5 ±4.7	138.1 ±1.4	0.761 ±0.001	15842 ±323	116704 ±436	**116617 ±436**	192 ±2
**GAR-105**	**GARTH**	105 ±2	301.1 ±0.3	416.8 ±8.4	133.4 ±1.7	0.763 ±0.001	9083 ±183	117953 ±505	**117859 ±506**	186 ±2
**GAR-140**	**GARTH**	140 ±2	280.1 ±0.3	375.8 ±7.5	135.4 ±1.5	0.771 ±0.001	9475 ±190	119895 ±459	**119802 ±460**	190 ±2
**AGAR-17**	**GARTH**	172 ±2	200.1 ±0.2	193.8 ±3.9	187.1 ±1.6	0.821 ±0.002	13975 ±283	122522 ±580	**122435 ±580**	264 ±2
**GAR-185**	**GARTH**	185 ±2	333.0 ±0.4	560.8 ±11.3	176.9 ±1.3	0.839 ±0.002	8215 ±166	129691 ±537	**129584 ±538**	255 ±2
**AGAR-20**	**GARTH**	205 ±2	251.3 ±0.3	167.4 ±3.4	173.1 ±1.4	0.834 ±0.002	20655 ±419	129233 ±554	**129153 ±554**	249 ±2
**AGAR-22**	**GARTH**	221 ±2	295.0 ±0.4	371.1 ±7.5	177.3 ±1.5	0.842 ±0.002	11035 ±223	130433 ±655	**130339 ±655**	256 ±2
**AGAR-24.5**	**GARTH**	238 ±2	378.8 ±0.5	1747 ±35	169.4 ±1.6	0.844 ±0.002	3015 ±61	132851 ±656	**132679 ±659**	246 ±2
**AGAR-26**	**GARTH**	262.5 ±2	339.9 ±0.6	628.0 ±12.6	167.1 ±1.7	0.851 ±0.002	7593 ±153	135594 ±792	**135486 ±792**	245 ±3
**GAR-276**	**GARTH**	276 ±2	333.1 ±0.4	241.6 ±4.9	152.9 ±1.6	0.846 ±0.002	19228 ±389	137635 ±694	**137554 ±694**	226 ±2
**GAR-326**	**GARTH**	326 ±2	355.9 ±0.5	395.7 ±7.9	161.0 ±1.6	0.857 ±0.002	12712 ±256	139076 ±704	**138985 ±704**	238 ±2
**GAR-364**	**GARTH**	364 ±2	346.0 ±0.3	112.5 ±2.3	137.8 ±1.4	0.843 ±0.001	42743 ±871	140838 ±598	**140769 ±598**	205 ±2
**GAR-392.5**	**GARTH**	392.5 ±2	261.7 ±0.3	117.4 ±2.4	123.3 ±1.4	0.834 ±0.001	30642 ±629	141891 ±634	**141813 ±634**	184 ±2
**GAR-412**	**GARTH**	412 ±2	301.6 ±0.2	221.6 ±4.5	110.4 ±1.4	0.826 ±0.001	18521 ±373	142923 ±567	**142844 ±567**	165 ±2
**GAR-437.5**	**GARTH**	437.5 ±2	337.3 ±0.4	795 ±16	103.8 ±1.3	0.827 ±0.002	5783 ±117	145398 ±859	**145271 ±860**	156 ±2
**NEI-118**	**NEITH**	118 ±2	47.34 ±0.05	884.5 ±17.7	302.7 ±1.5	0.944 ±0.002	833 ±17	130383 ±552	**129944 ±612**	437 ±2
**NEI-118**	**NEITH**	118 ±2	44.9 ±0.2	73 ±2	299.4 ±5.4	0.924 ±0.005	9341 ±263	126255 ±1688	**126195 ±1688**	428 ±8
**NEI-119.5**	**NEITH**	119.5 ±2	37.18 ±0.04	45.4 ±1.2	290.2 ±1.6	0.928 ±0.002	12531 ±341	129015 ±633	**128921 ±633**	418 ±2
**NEI-122**	**NEITH**	122 ±2	44.8 ±0.1	138.5 ±2.8	283.4 ±1.9	0.921 ±0.002	4906 ±100	128543 ±645	**128419 ±647**	407 ±3
**NEI-126**	**NEITH**	126 ±2	416.1 ±0.6	3410.5 ±68.4	266.4 ±2.1	0.905 ±0.002	1821 ±37	128019 ±738	**127786 ±746**	382 ±3
**NEI-127**	**NEITH**	127 ±2	43.3 ±0.1	122.1 ±2.5	250.6 ±1.9	0.906 ±0.002	5299 ±111	131540 ±648	**131411 ±649**	363 ±3
**NEI-130**	**NEITH**	130 ±2	39.55 ±0.04	317.7 ±6.4	262.9 ±1.5	0.905 ±0.002	1857 ±37	128692 ±538	**128462 ±551**	378 ±2
**NEI-132**	**NEITH**	132 ±2	30.9 ±0.1	319.9 ±3.3	233.9 ±6.0	0.904 ±0.008	1439 ±18	134780 ±2619	**134495 ±2615**	342 ±9
**NEI-134**	**NEITH**	134 ±2	35.87 ±0.03	218.3 ±4.4	222.9 ±1.5	0.895 ±0.002	2426 ±49	134972 ±633	**134778 ±639**	326 ±2
**NEI-136**	**NEITH**	136 ±2	33.52 ±0.02	521.1 ±10.5	235.4 ±1.2	0.895 ±0.002	949 ±19	131915 ±589	**131509 ±634**	341 ±2
**NEI-138**	**NEITH**	138 ±2	39.45 ±0.04	906.3 ±18.2	267.9 ±1.6	0.946 ±0.002	679 ±14	138715 ±661	**138175 ±741**	396 ±3
**NEI-138.5**	**NEITH**	138.5 ±2	34.9 ±0.2	1712.8 ±8.3	323.1 ±6.8	0.99 ±0.01	333 ±4	138455 ±3170	**137424 ±3178**	476 ±11

Analytical errors are 2s of the mean.

Corrected ^{230}Th ages assume the initial $^{230}Th/^{232}Th$ atomic ratio of 4.4 ± 2.2 x10^{-6}. Those are the values for a material at secular equilibrium, with the bulk earth $^{232}Th/^{238}U$ value of 3.8. The errors are arbitrarily assumed to be 50%.

U decay constants: $\lambda_{238} = 1.55125 \times 10^{-10}$ and $\lambda_{234} = 2.82206 \times 10^{-6}$. Th decay constant: $\lambda_{230} = 9.1705 \times 10^{-6}$.

[a] $\delta^{234}U = ([^{234}U/^{238}U]_{activity} - 1) \times 1000$.

[b] $[^{230}Th/^{238}U]_{activity} = 1 - e^{-l230 * T} + (d^{234}U_{measured}/1000)[l230/(l230 - l234)](1 - e^{-(l230 - l234)T})$, where T is the age.

[c] The degree of detrital ^{230}Th contamination is indicated by the $[^{230}Th/^{232}Th]$ atomic ratio instead of the activity ratio.

[d] Age corrections were calculated using an average crustal $^{230}Th/^{232}Th$ atomic ratio of $4.4 \times 10^{-6} \pm 2.2 \times 10^{-6}$.

[e] B.P. stands for "Before Present" where the "Present" is defined as the year 1950 A.D.

Supplementary Table 6.

^{230}Th dating results for stalagmites spanning TII.

1 Genty, D. *et al.* Calculation of past dead carbon proportion and variability by the comparison of AMS 14 C and TIMS U/Th ages on two Holocene stalagmites. *Radiocarbon* **41**, 251-270 (1999).

2 Schmidt, M. W., Spero, H. J. & Lea, D. W. Links between salinity variation in the Caribbean and North Atlantic thermohaline circulation. *Nature* **428**, 160 (2004).

3 Lambeck, K., Rouby, H., Purcell, A., Sun, Y. & Sambridge, M. Sea level and global ice volumes from the Last Glacial Maximum to the Holocene. *Proceedings of the National Academy of Sciences* **111**, 15296-15303 (2014).

4 Skinner, L. & Shackleton, N. Deconstructing Terminations I and II: revisiting the glacioeustatic paradigm based on deep-water temperature estimates. *Quaternary Science Reviews* **25**, 3312-3321 (2006).

5 Català, A., Cacho, I., Frigola, J., Pena, L. D. & Lirer, F. Holocene hydrography evolution in the Alboran Sea: a multi-record and multi-proxy comparison. *Climate of the Past* **15**, 927-942 (2019).

6 Siani, G., Magny, M., Paterne, M., Debret, M. & Fontugne, M. Paleohydrology reconstruction and Holocene climate variability in the South Adriatic Sea. *Climate of the Past* **9**, 499-515 (2013).

7 Sicre, M.-A., Siani, G., Genty, D., Kallel, N. & Essallami, L. Seemingly divergent sea surface temperature proxy records in the central Mediterranean during the last deglaciation. *Climate of the Past* **9**, 1375-1383 (2013).

8 Peck, V. L. *et al.* High resolution evidence for linkages between NW European ice sheet instability and Atlantic Meridional Overturning Circulation. *Earth and Planetary Science Letters* **243**, 476-488 (2006).

9 Genty, D. *et al.* Timing and dynamics of the last deglaciation from European and North African δ13C stalagmite profiles—comparison with Chinese and South Hemisphere stalagmites. *Quaternary Science Reviews* **25**, 2118-2142 (2006).

10 Baldini, L. M. *et al.* North Iberian temperature and rainfall seasonality over the Younger Dryas and Holocene. *Quaternary Science Reviews* **226**, 105998 (2019).

11 Zanchetta, G. *et al.* Coeval dry events in the central and eastern Mediterranean basin at 5.2 and 5.6 ka recorded in Corchia (Italy) and Soreq caves (Israel) speleothems. *Global and Planetary Change* **122**, 130-139 (2014).

12 Belli, R. *et al.* Regional climate variability and ecosystem responses to the last deglaciation in the northern hemisphere from stable isotope data and calcite fabrics in two northern Adriatic stalagmites. *Quaternary Science Reviews* **72**, 146-158 (2013).

13 Bar-Matthews, M., Ayalon, A. & Kaufman, A. Late Quaternary paleoclimate in the eastern Mediterranean region from stable isotope analysis of speleothems at Soreq Cave, Israel. *Quaternary Research* **47**, 155-168 (1997).

14 Grant, K. *et al.* Rapid coupling between ice volume and polar temperature over the past 150,000 years. *Nature* **491**, 744 (2012).

15 Almogi-Labin, A. *et al.* Climatic variability during the last∼ 90 ka of the southern and northern Levantine Basin as evident from marine records and speleothems. *Quaternary Science Reviews* **28**, 2882-2896 (2009).

16 Ausín, B. *et al.* (In) coherent multiproxy signals in marine sediments: Implications for high-resolution paleoclimate reconstruction. *Earth and Planetary Science Letters* **515**, 38-46 (2019).

17 Cacho, I. *et al.* Dansgaard-Oeschger and Heinrich event imprints in Alboran Sea paleotemperatures. *Paleoceanography* **14**, 698-705 (1999).

18 Stoll, H. *et al.* Interpretation of orbital scale variability in mid-latitude speleothem δ 18 O: Significance of growth rate controlled kinetic fractionation effects. *Quaternary Science Reviews* **127**, 215-228 (2015).
19 Martrat, B. *et al.* Four climate cycles of recurring deep and surface water destabilizations on the Iberian margin. *Science* **317**, 502-507 (2007).
20 de Abreu, L., Shackleton, N. J., Schönfeld, J., Hall, M. & Chapman, M. Millennial-scale oceanic climate variability off the Western Iberian margin during the last two glacial periods. *Marine Geology* **196**, 1-20 (2003).
21 Voelker, A. H. & de Abreu, L. A review of abrupt climate change events in the Northeastern Atlantic Ocean (Iberian Margin): Latitudinal, longitudinal, and vertical gradients. *Abrupt Climate Change: Mechanisms, Patterns, and Impacts* **193**, 15-37 (2011).
22 Torner, J. *et al.* Ocean-atmosphere interconnections from the last interglacial to the early glacial: An integration of marine and cave records in the Iberian region. *Quaternary Science Reviews* **226**, 106037 (2019).
23 Martrat, B. *et al.* Abrupt temperature changes in the Western Mediterranean over the past 250,000 years. *Science* **306**, 1762-1765 (2004).
24 Jiménez-Amat, P. & Zahn, R. Offset timing of climate oscillations during the last two glacial-interglacial transitions connected with large-scale freshwater perturbation. *Paleoceanography* **30**, 768-788 (2015).
25 Tzedakis, P. *et al.* Enhanced climate instability in the North Atlantic and southern Europe during the Last Interglacial. *Nature communications* **9**, 4235 (2018).
26 Mokeddem, Z., McManus, J. F. & Oppo, D. W. Oceanographic dynamics and the end of the last interglacial in the subpolar North Atlantic. *Proceedings of the National Academy of Sciences* **111**, 11263-11268 (2014).
27 Barker, S. *et al.* 800,000 years of abrupt climate variability. *science* **334**, 347-351 (2011).
28 Irvalı, N. *et al.* Rapid switches in subpolar North Atlantic hydrography and climate during the Last Interglacial (MIS 5e). *Paleoceanography* **27** (2012).
29 Irvalı, N. *et al.* Evidence for regional cooling, frontal advances, and East Greenland Ice Sheet changes during the demise of the last interglacial. *Quaternary Science Reviews* **150**, 184-199 (2016).
30 Galaasen, E. V. *et al.* Rapid reductions in North Atlantic deep water during the peak of the last interglacial period. *Science* **343**, 1129-1132 (2014).
31 Nicholl, J. A. *et al.* A Laurentide outburst flooding event during the last interglacial period. *Nature Geoscience* **5**, 901 (2012).
32 Martrat, B., Jimenez-Amat, P., Zahn, R. & Grimalt, J. O. Similarities and dissimilarities between the last two deglaciations and interglaciations in the North Atlantic region. *Quaternary Science Reviews* **99**, 122-134 (2014).
33 Yokoyama, Y. *et al.* Rapid glaciation and a two-step sea level plunge into the Last Glacial Maximum. *Nature* **559**, 603-607 (2018).
34 Gherardi, J.-M. *et al.* Evidence from the Northeastern Atlantic basin for variability in the rate of the meridional overturning circulation through the last deglaciation. *Earth and Planetary Science Letters* **240**, 710-723 (2005).
35 Skinner, L., Elderfield, H. & Hall, M. Phasing of millennial climate events and Northeast Atlantic deep-water temperature change since 50 ka BP. *Washington DC American Geophysical Union Geophysical Monograph Series* **173**, 197-208 (2007).
36 Skinner, L. & Shackleton, N. Rapid transient changes in northeast Atlantic deep water ventilation age across Termination I. *Paleoceanography* **19** (2004).
37 Chalk, T. B. *et al.* Causes of ice age intensification across the Mid-Pleistocene Transition. *Proceedings of the National Academy of Sciences* **114**, 13114-13119 (2017).
